# Supplementary material for: Loss of RASGRP1 in humans impairs T‐cell expansion leading to Epstein‐Barr virus susceptibility
Source: EMBO Mol Med. 2018 Jan 8;10(2):188–99. doi: 10.15252/emmm.201708292 (PMC5801500; doi:10.15252/emmm.201708292)
Supplement: Supplementary file 1 — Expanded View Figures PDF [file EMMM-10-188-s001.pdf]

Expanded View Figures

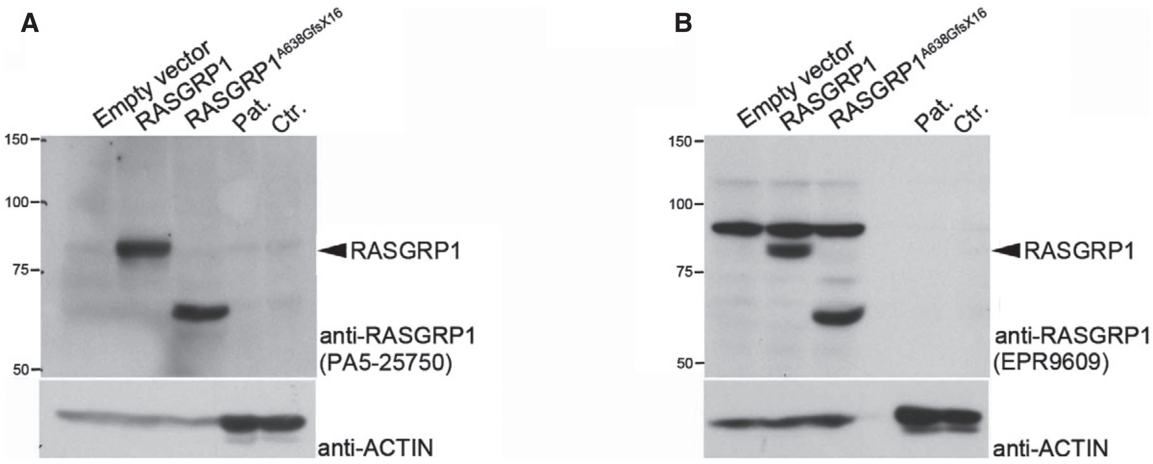

Figure EV1. RASGRP1 expression is not detected in T-cell blasts with the anti-RASGRP1 antibodies PA5-25750 and EPR9609.

Comparison of RASGRP1 expression in T-cell blasts of a healthy control (Ctr.) and P1.1 (Pat.) and in HEK293T cells transfected with empty vector, WT-RASGRP1 or $RASGRP1^{A638GfsX16}$.

A RASGRP1 detection using the anti-RASGRP1 antibody PA5-25750. Actin was used as a loading control. Detection of RASGRP1 required long exposure (1 h to overnight).
B RASGRP1 detection using the anti-RASGRP1 antibody EPR9609. Actin was used as a loading control. Detection of RASGRP1 required long exposure (1 h to overnight).

Data information: One representative of three independent experiments.

Source data are available online for this figure.

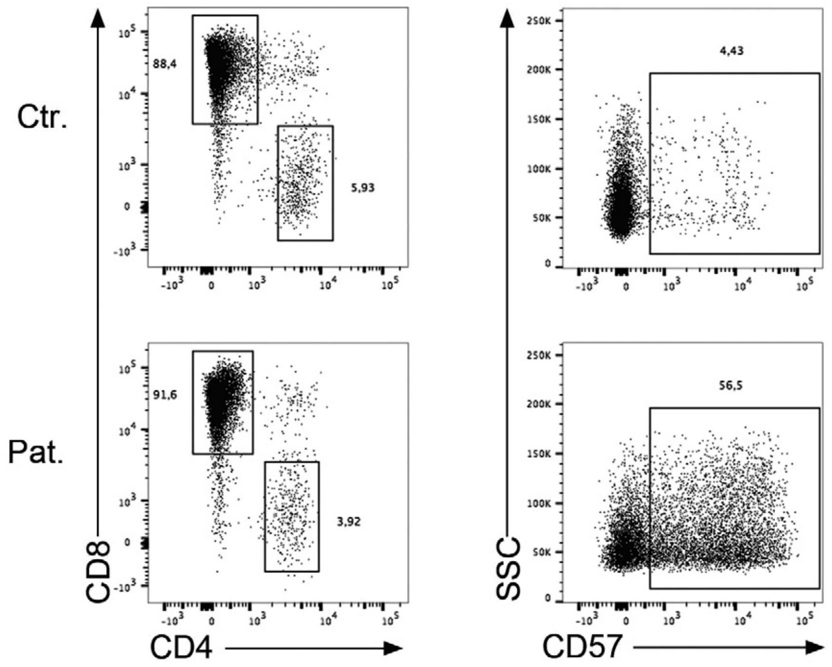

Figure EV2. Accumulation of $CD57^{+}$ senescent T cells in RASGRP1-deficient T-cell blasts after 12–15 days of culture.

Left panel: Dot plots showing the staining of CD4 and CD8 on T-cell blasts at day 20 of culture from a healthy control (Ctr.) and P1.1 (Pat.). Right panel: Dot plots showing the staining for CD57 and the SSC gated on CD8-positive cells. One representative experiment of four cultures from different blood samples.

© 2017 The Authors

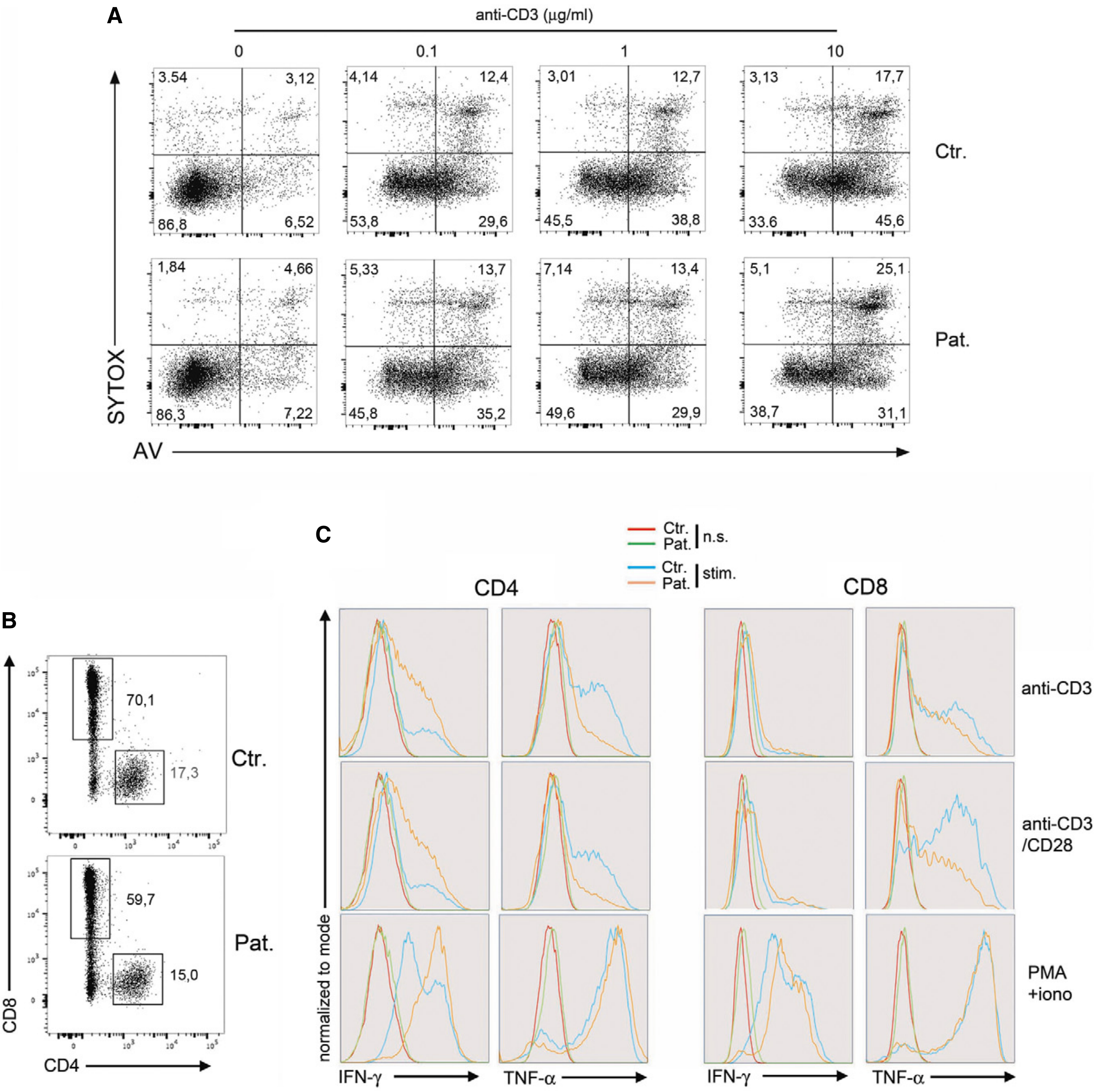

Figure EV3. Activation-induced cell death and cytokine production of T-cell blasts from patient P1.1.

A Normal activation-induced cell death (AICD) in RASGRP1-deficient T cells. Representative dot plots showing apoptotic cells of a healthy control (Ctr.) and P1.1 (Pat.) detected by Annexin V (AV) and SYTOX staining after stimulation with the indicated concentration of anti-CD3 antibody for 12 h. Apoptotic and necrotic cells correspond to SYTOX$^-$AV$^+$ and SYTOX$^+$AV$^+$, respectively. One representative of two independent experiments.

B, C Intracellular TNF-α and IFN-γ production in RASGRP1-deficient T-cell blasts. (B) Dot plots of CD8 and CD4 staining on gated CD3$^+$ T-cell blasts for cytokine production. (C) FACS histograms of intracellular IFN-γ and TNF-α production on gated CD4$^+$ or CD8$^+$ T-cell blasts of a control donor (Ctr.) and P1.1 (Pat.) stimulated (stim.) or not (n.s.) for 12 h with coated anti-CD3 antibody, anti-CD3+CD28 beads, and PMA+ionomycin. One representative of two independent experiments.

 © 2017 The Authors

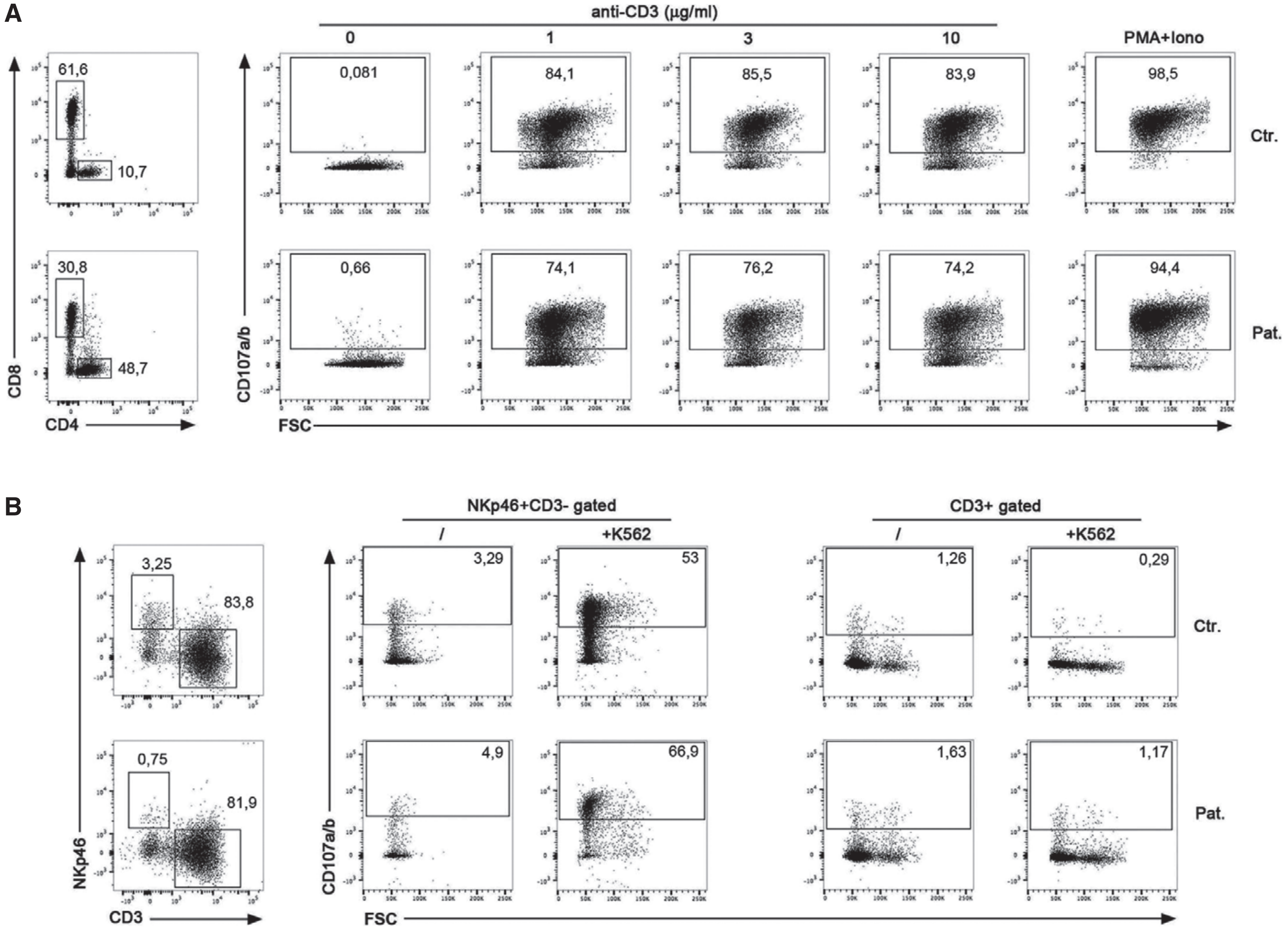

Figure EV4. Degranulation of RASGRP1-deficient CD8$^+$ T and NK cells.

A Representative dot plots showing the cell-surface expression of CD107a/b on gated CD8$^+$ T-cell blasts of a healthy control (Ctr.) and P1.1 (Pat.) that have been stimulated for 3 h with incremental doses of coated anti-CD3 antibody or PMA+ionomycin. One representative of three independent experiments.

B Representative dot plots showing the cell-surface expression of CD107a/b on gated NKp46$^+$CD3$^-$ cells corresponding to NK cells or CD3$^+$ cells of a healthy control (Ctr.) and P1.1 (Pat.) that have been stimulated for 3 h in the presence or not of K562 target cells. One representative of two independent experiments.

© 2017 The Authors

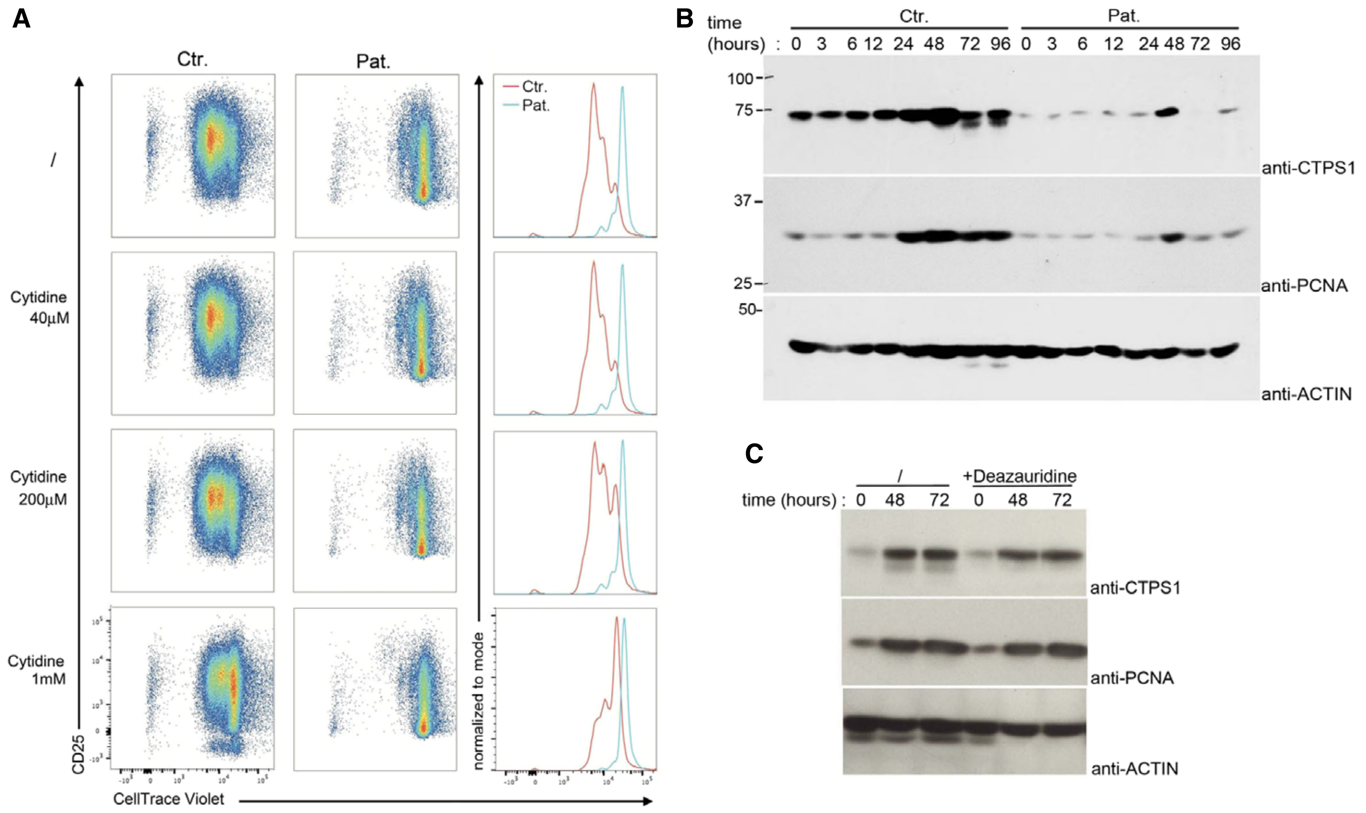

Figure EV5. Cytidine complementation of RASGRP1-deficient T-cell blasts and defect in PCNA expression in activated RASGRP1-deficient T cells.

A Cytidine complementation does not restore proliferation of RASGRP1 deficient T-cell blasts. Representative dot plots showing cell divisions by dilution of the CellTrace violet dye and expression of CD25 of control donor (Ctr.) or P1.1 (Pat.) T-cell blasts stimulated with anti-CD3 antibody in a medium containing no (/) or various concentrations of cytidine (40 µM, 200 µM, or 1 mM). Each peak of the histograms corresponds to a cell division. Right panels represent histograms from the dot plots of patient and control that have been overlaid. Data are representative of one of two independent experiments.

B Immunoblots for CTPS1 and PCNA expression of control donor (Ctr.) and P1.1 T-cell blasts (Pat.) stimulated with anti-CD3/CD28 antibodies for different periods of time. Actin was used as a loading control. One representative of two independent experiments from different blood samples.

C Control donor T-cell blasts stimulated or not for various periods of times with anti-CD3/CD28 beads in the presence or not (/) of 40 µM of deazauridine. Immunoblots of CTPS1 and PCNA expression. Actin was used as a loading control. One representative of two independent experiments from different blood samples.

Source data are available online for this figure.

© 2017 The Authors
