# Supplementary material for: Loss of RASGRP1 in humans impairs T‐cell expansion leading to Epstein‐Barr virus susceptibility
Source: EMBO Mol Med. 2018 Jan 8;10(2):188–99. doi: 10.15252/emmm.201708292 (PMC5801500; doi:10.15252/emmm.201708292)
Supplement: Supplementary file 2 — Source Data for Expanded View [file EMMM-10-188-s007.zip › Source_data_EV5.pdf]

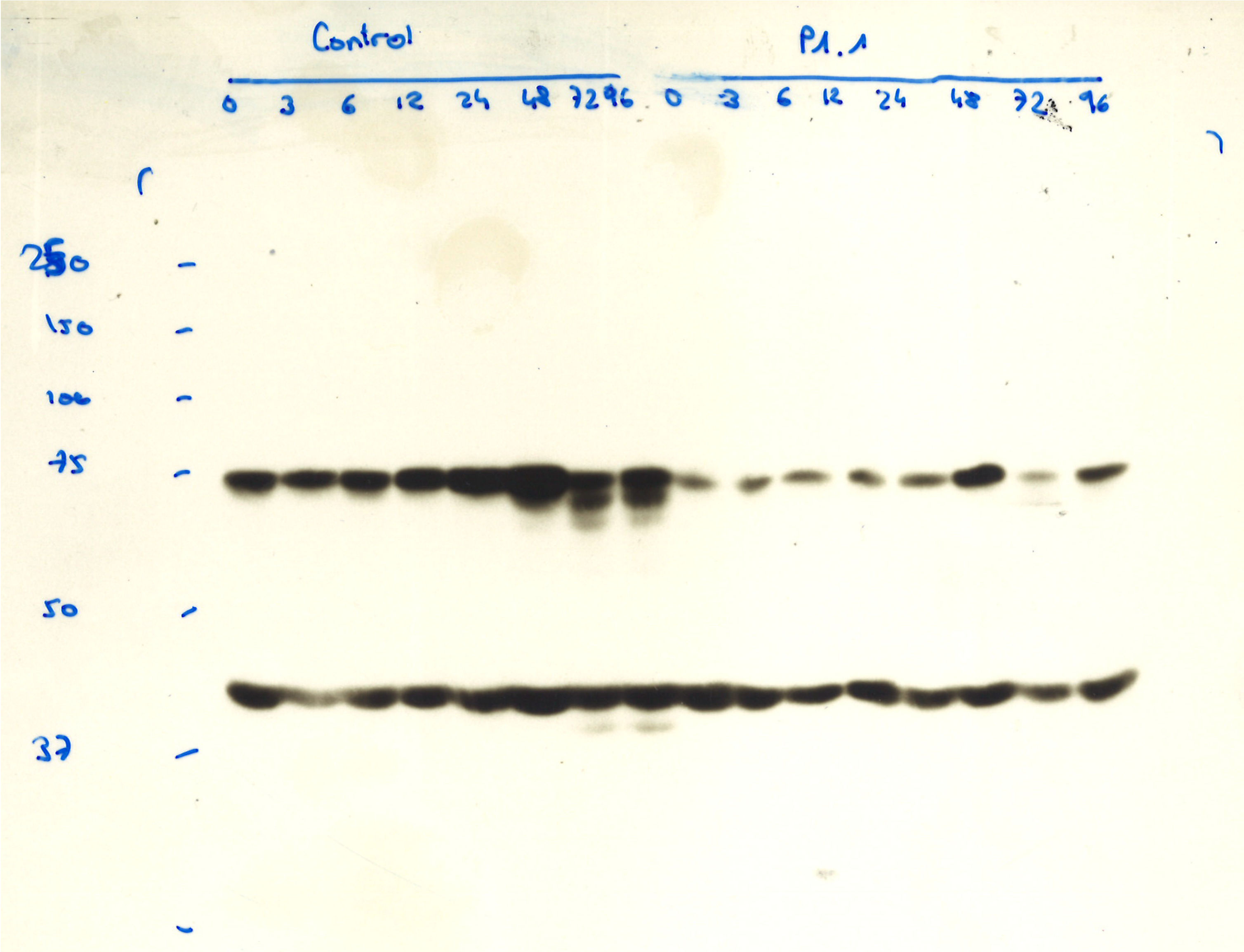

Figure EV5 Panel B anti-CTPS1 (Upper) anti-ACTIN (Lower)

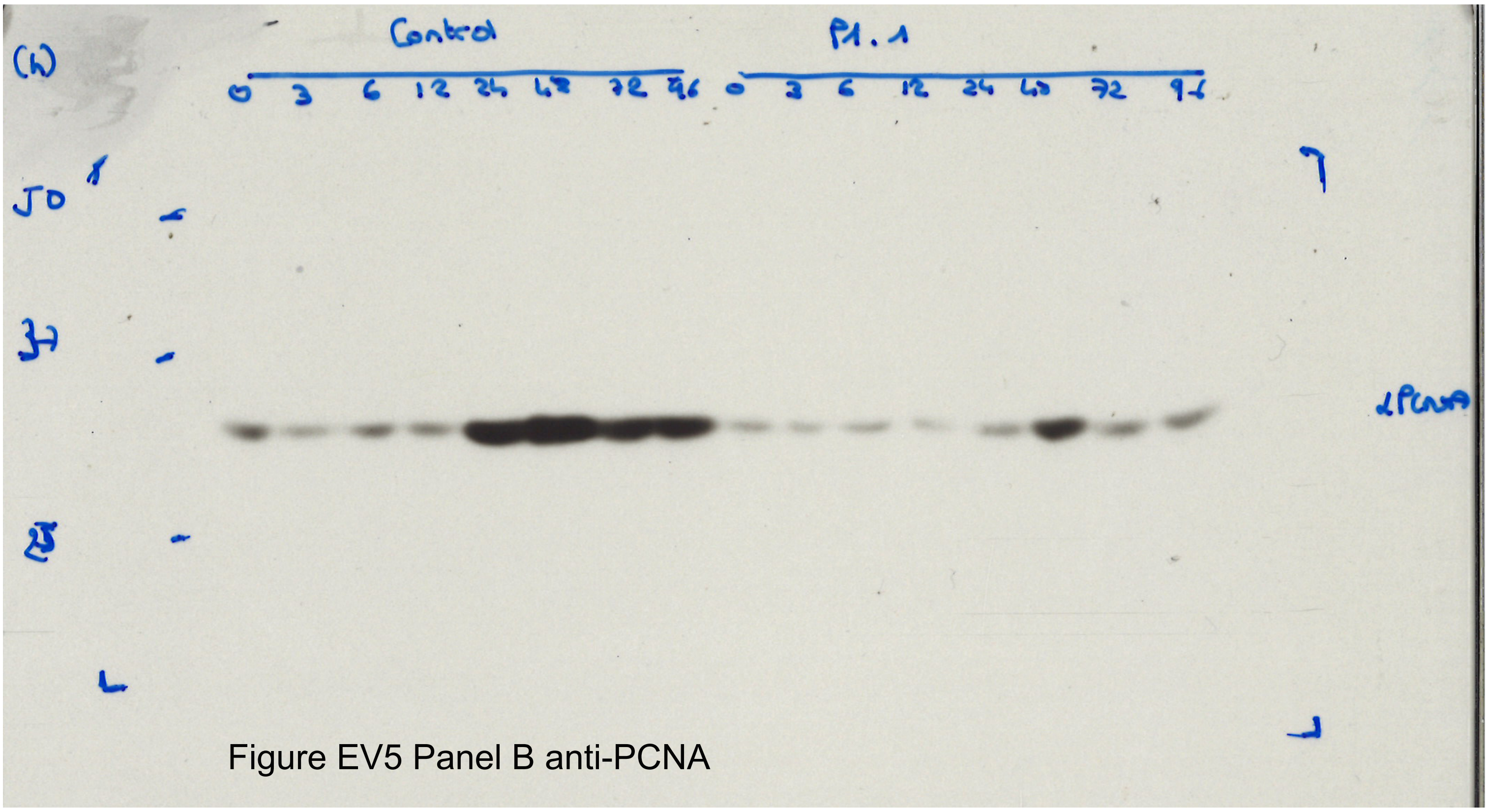

Figure EV5 Panel B anti-PCNA

Figure EV5 Panel C anti-CTPS1 (Upper) anti-ACTIN (Lower)

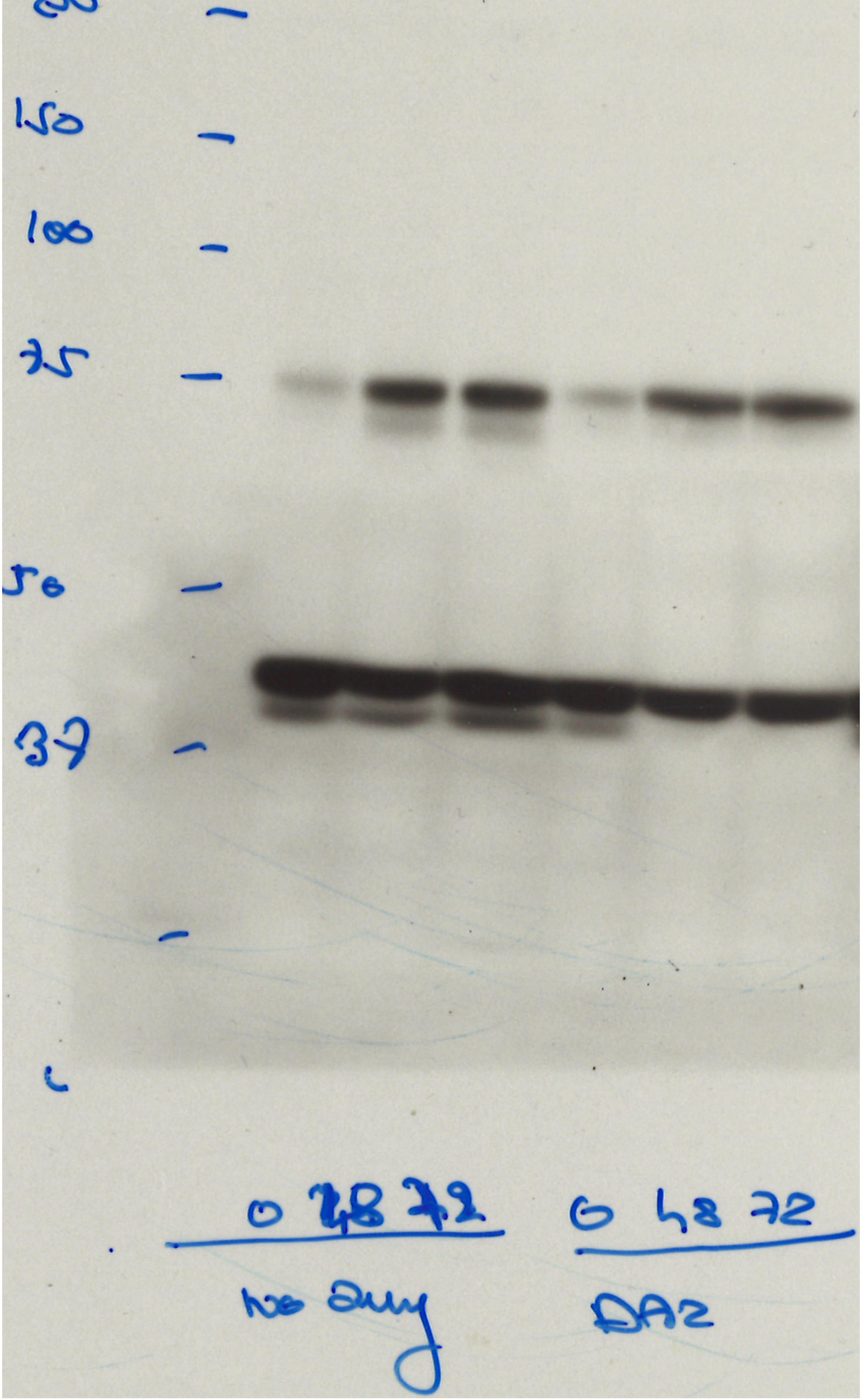

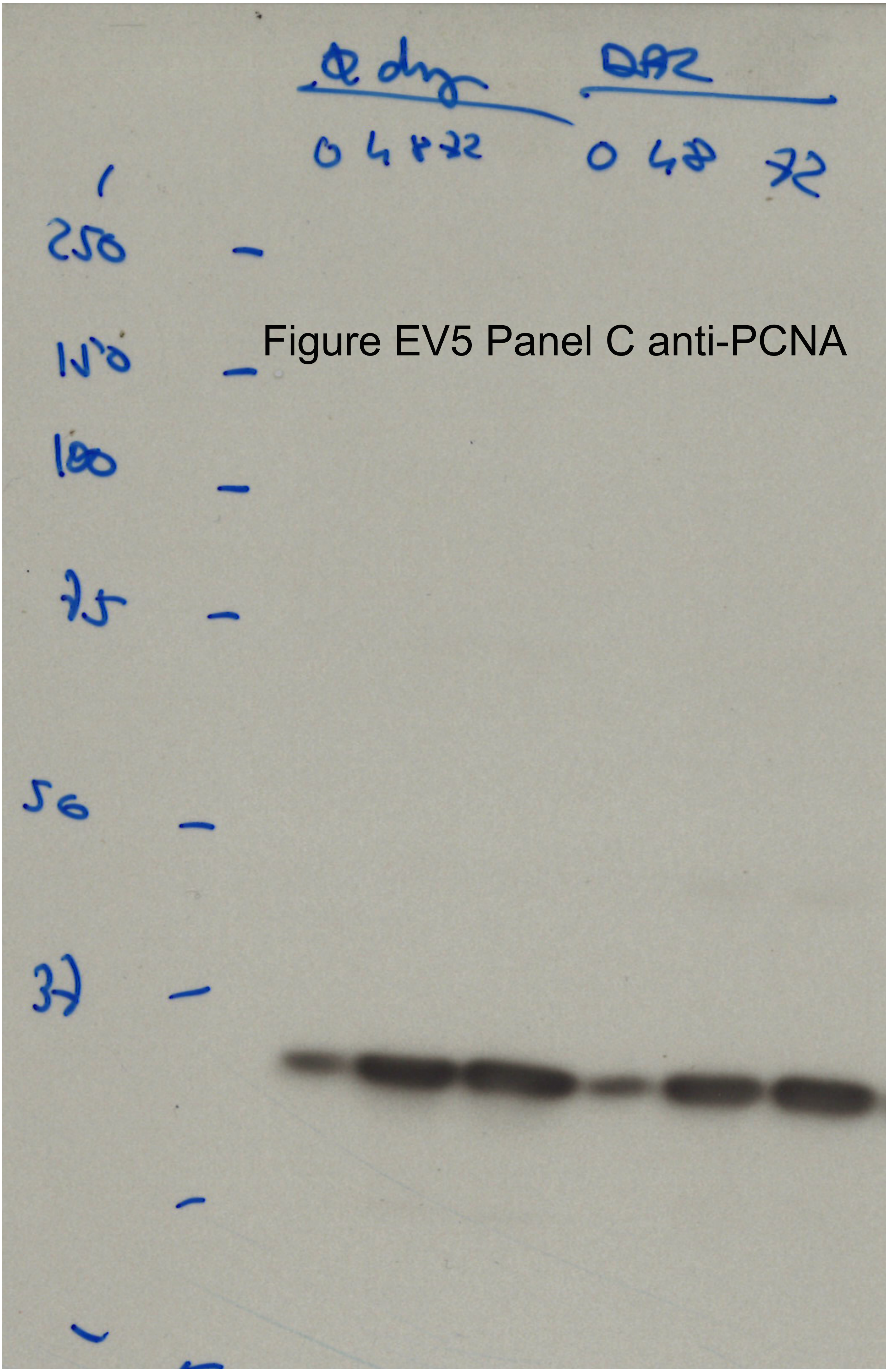

Figure EV5 Panel C anti-PCNA
