# Supplementary figures and images for: Loss of RASGRP1 in humans impairs T‐cell expansion leading to Epstein‐Barr virus susceptibility
Source: EMBO Mol Med. 2018 Jan 8;10(2):188–99. doi: 10.15252/emmm.201708292 (PMC5801500; doi:10.15252/emmm.201708292)

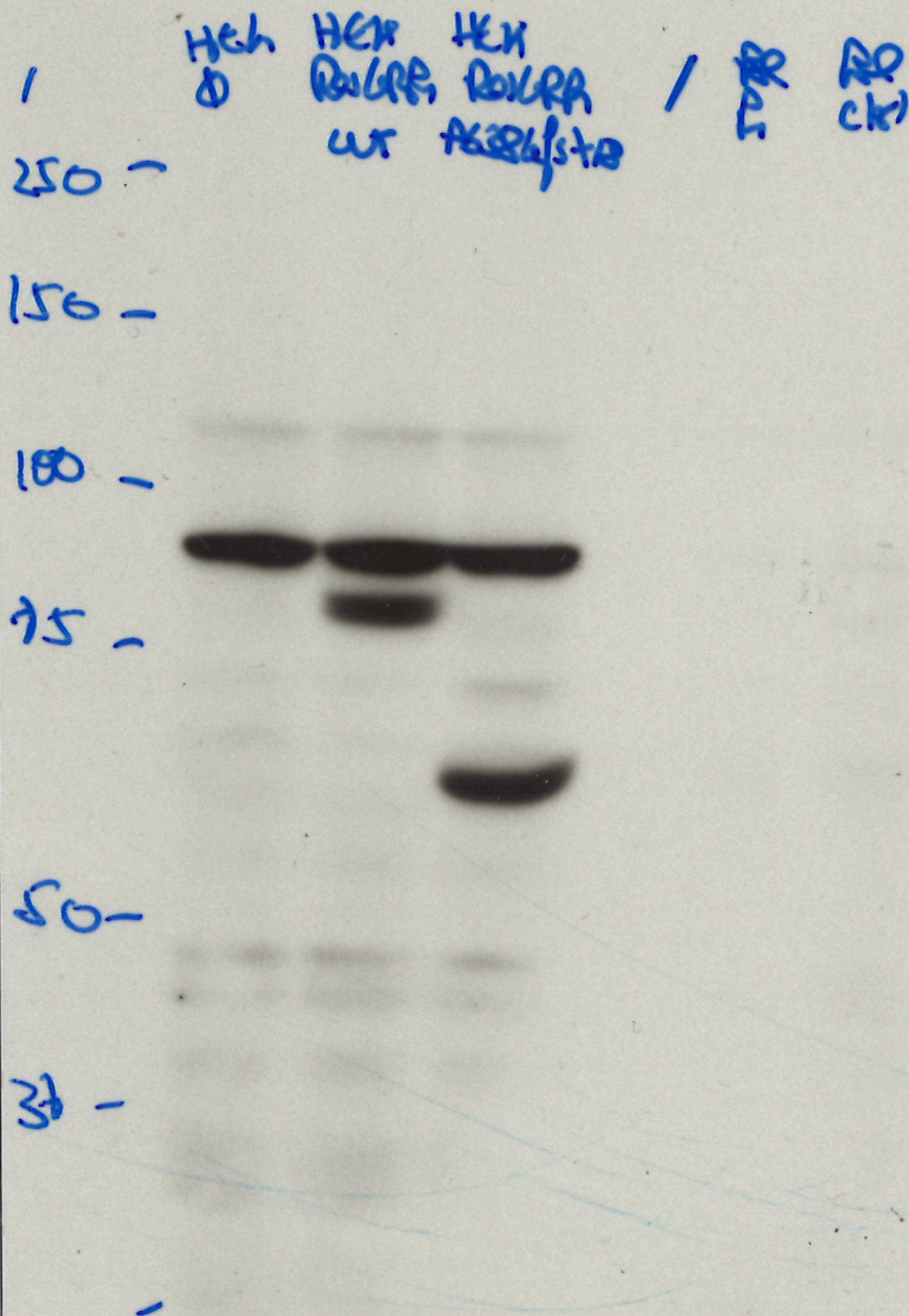

Figure EV1 Panel B anti-RASGRP1

FigEV1 Panel B anti-ACTIN

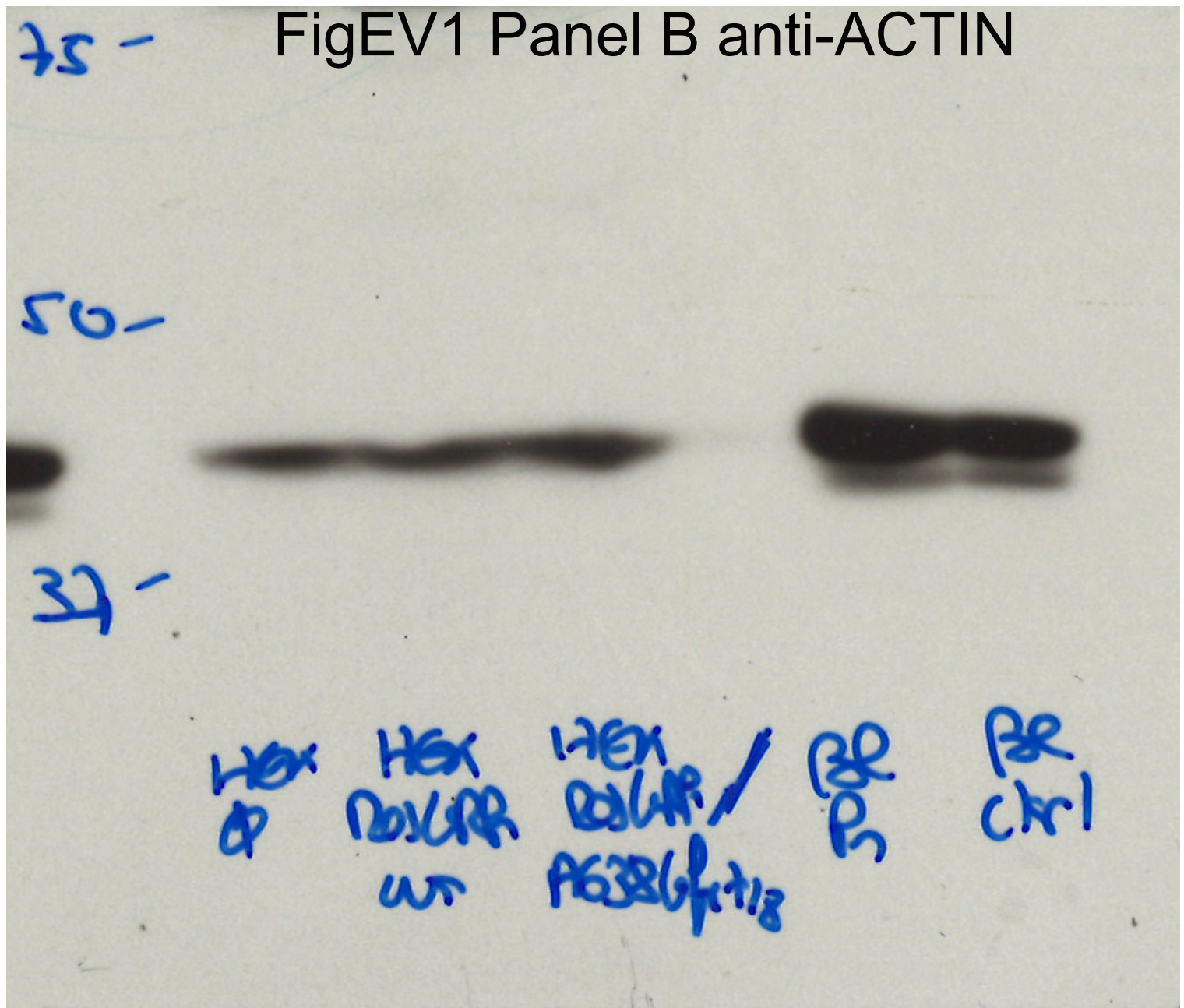

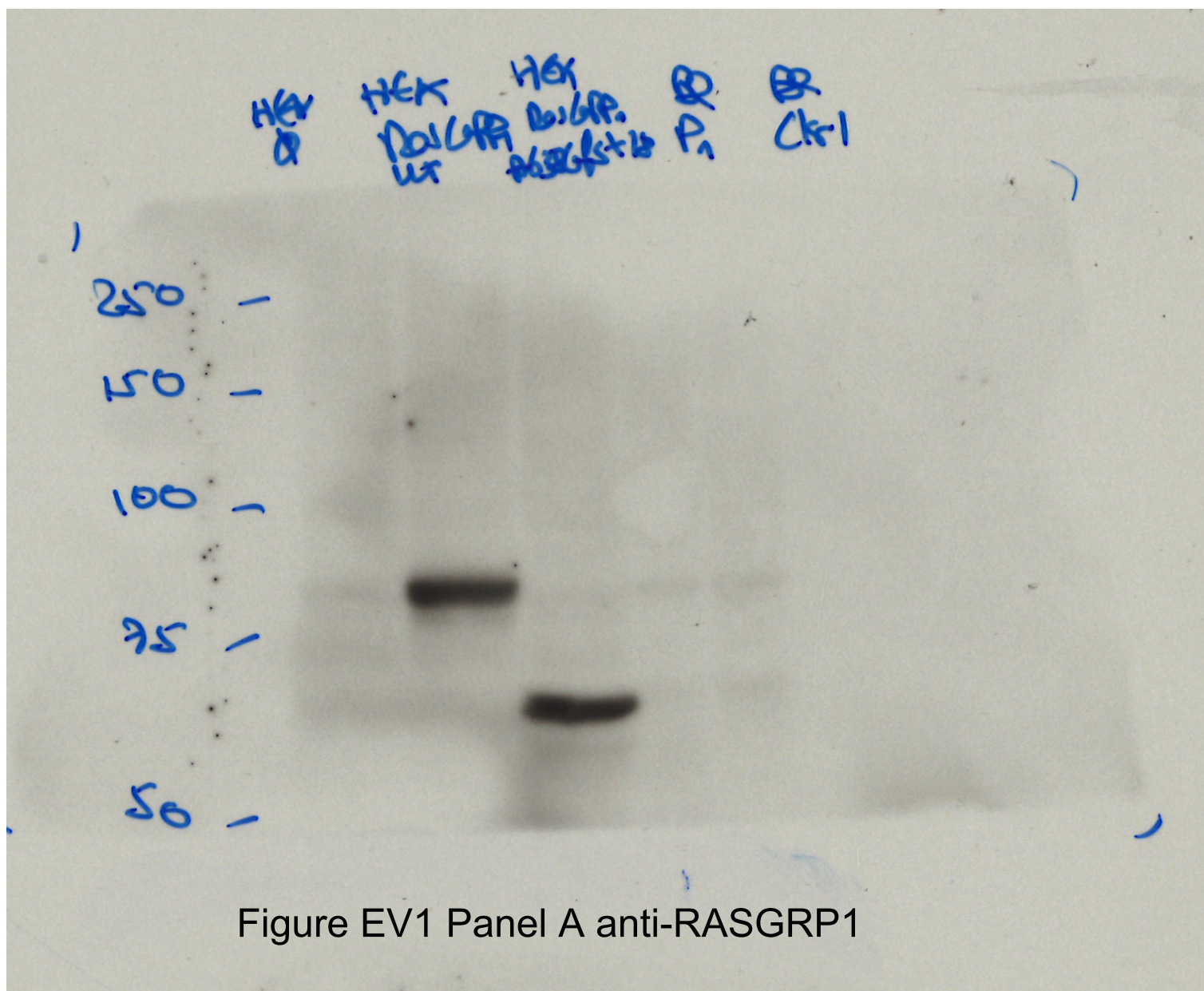

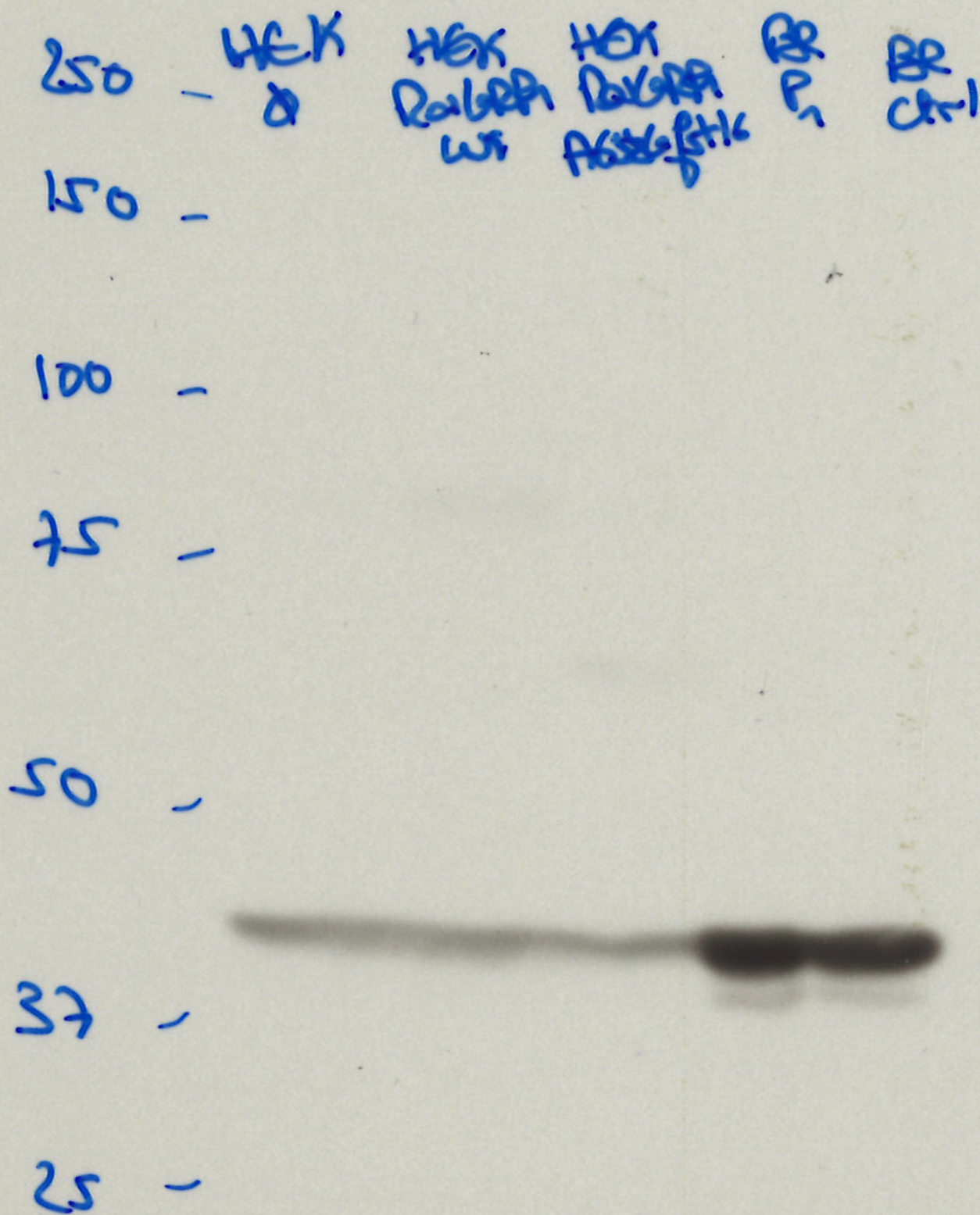

Figure EV1 Panel A anti-ACTIN

Supplement: Supplementary file 2 — Source Data for Expanded View [file EMMM-10-188-s007.zip › Source_Data_EV1.pdf]

Figure 3 Panel C anti-RASGRP1 (upper)  
anti-ACTIN (lower)

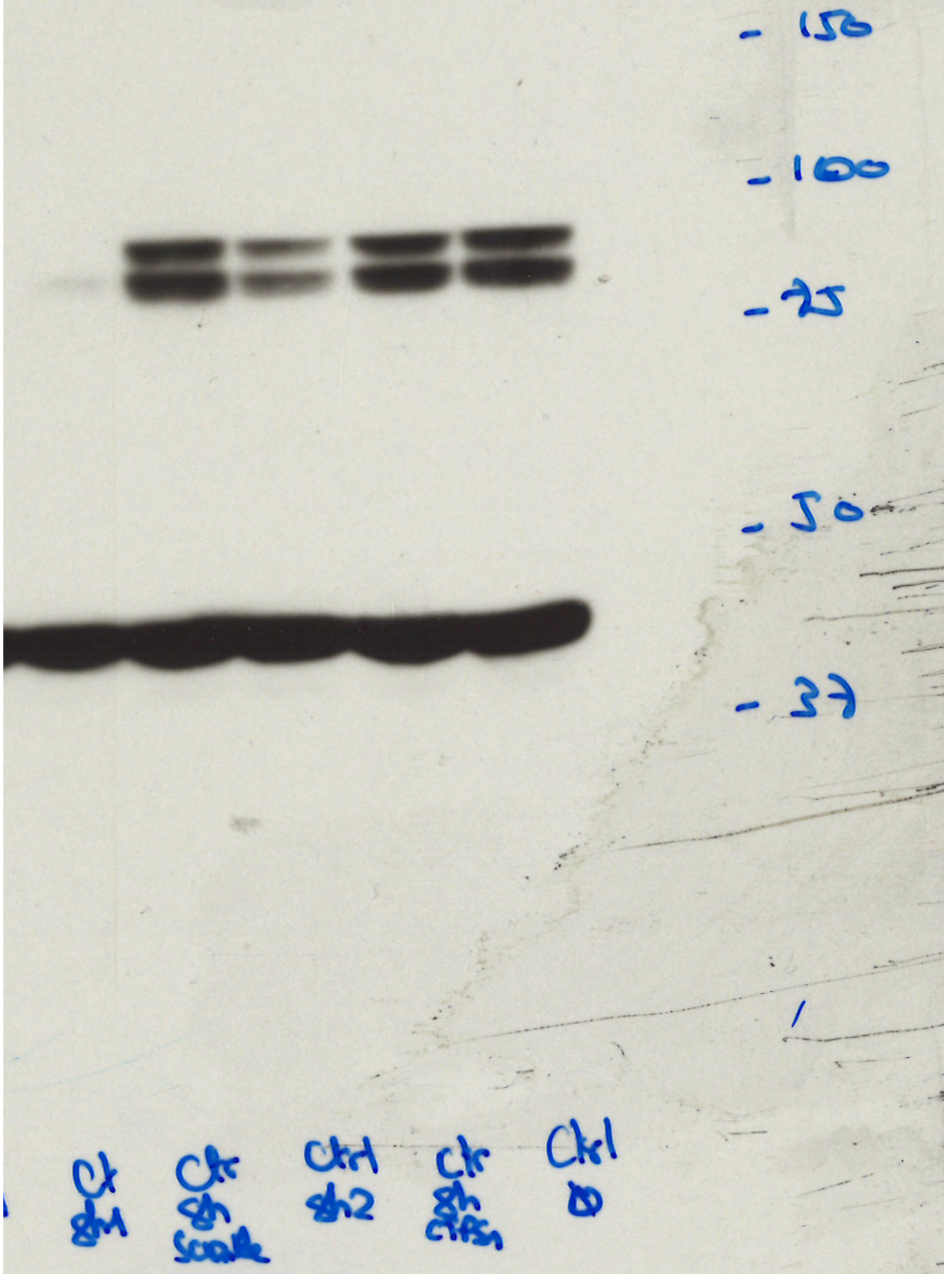

Figure 3 Panel C anti-ACTIN

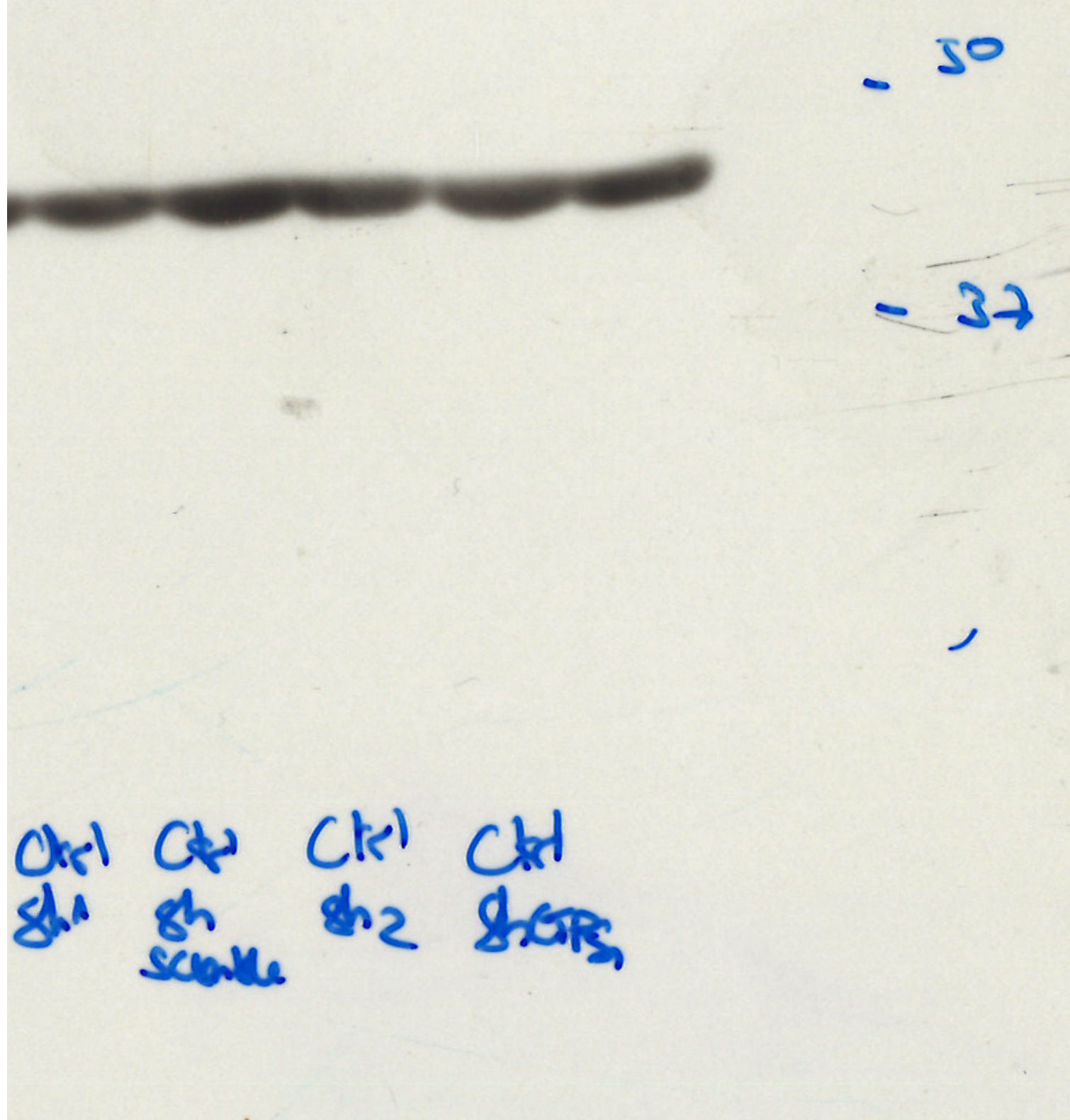

Supplement: Supplementary file 6 — Source Data for Figure 3 [file EMMM-10-188-s004.pdf]

Figure 5 Panel A anti-RASGRP1 (upper)  
anti-ACTIN (lower)

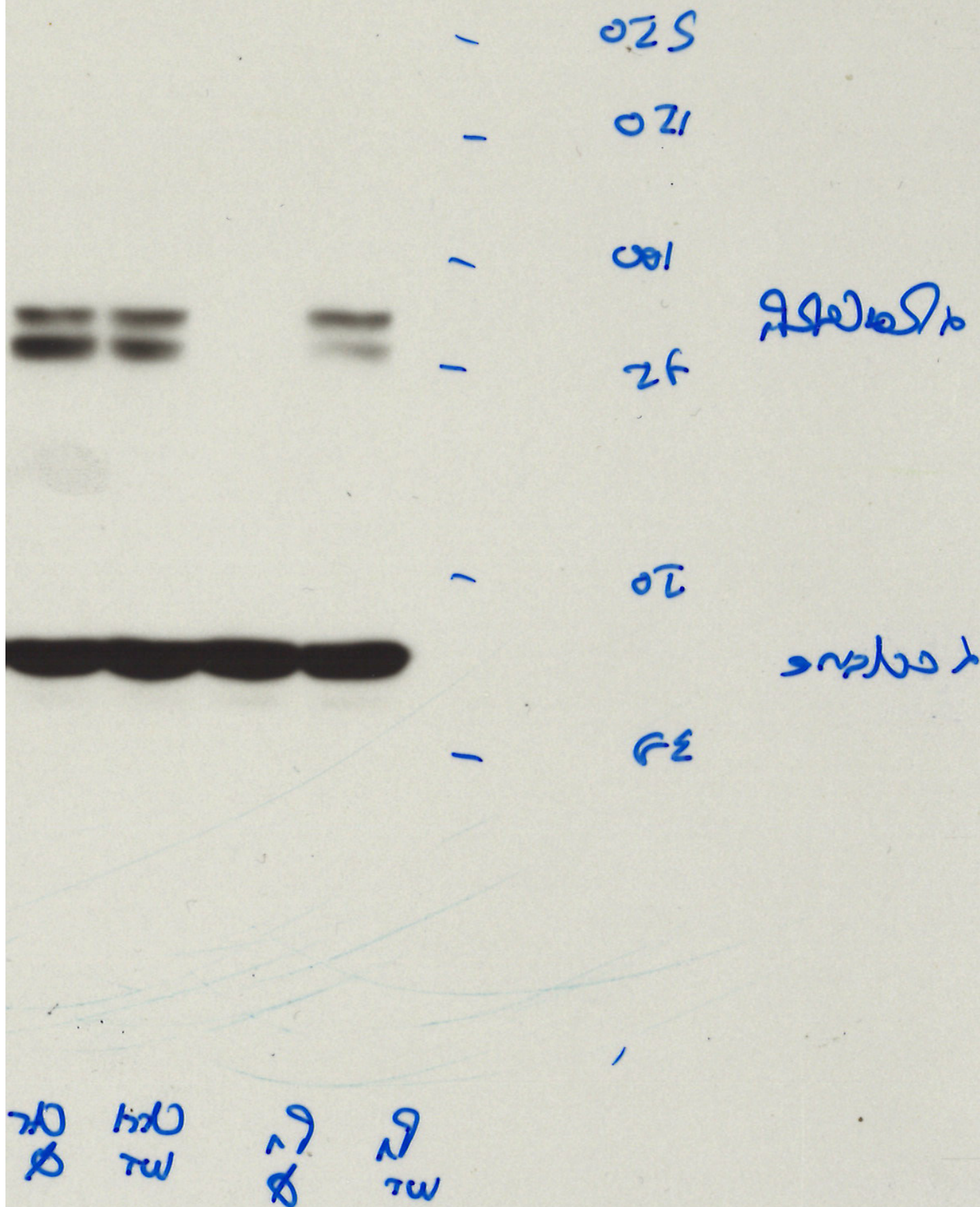

Supplement: Supplementary file 7 — Source Data for Figure 5 [file EMMM-10-188-s005.pdf]

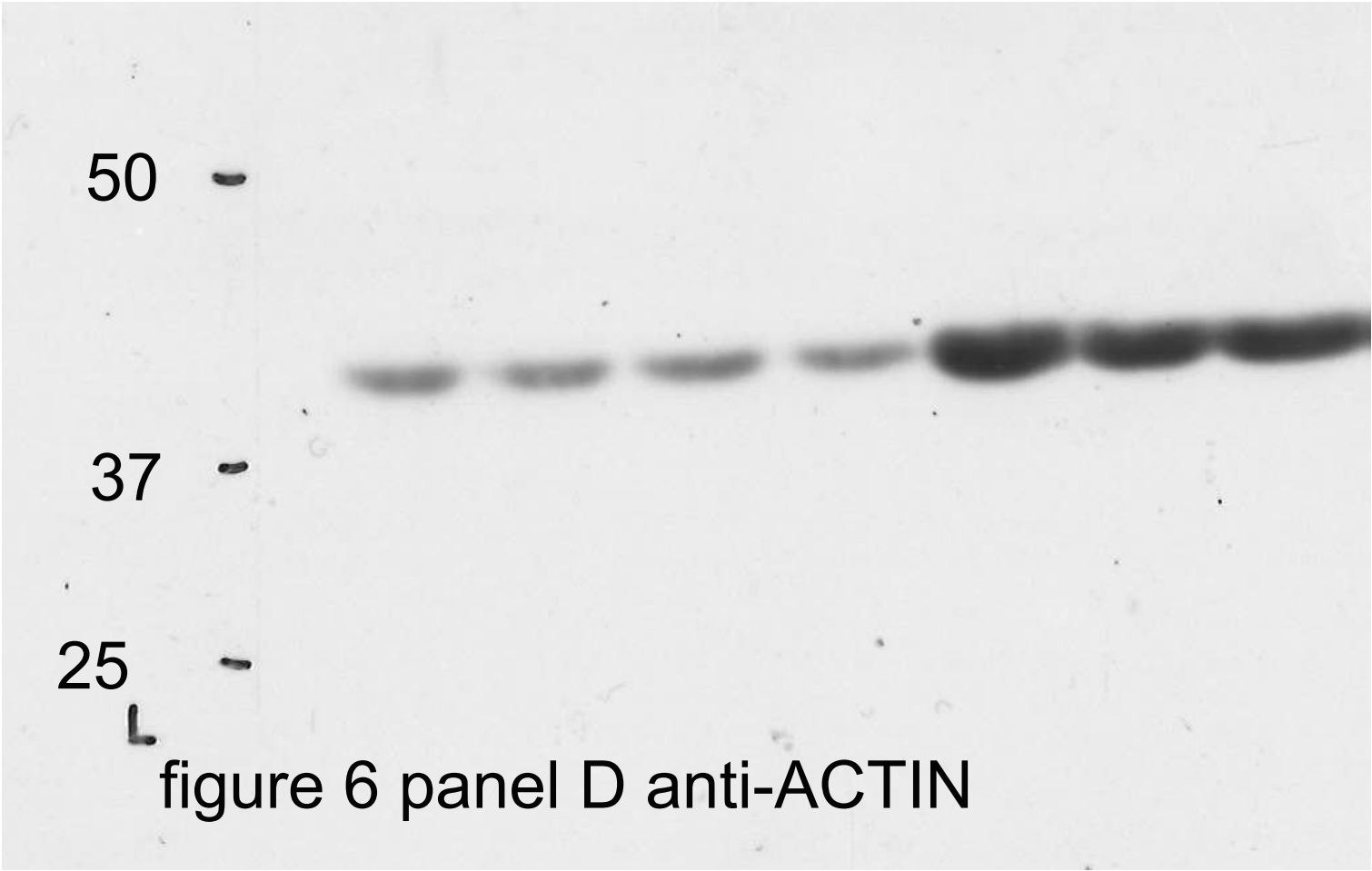

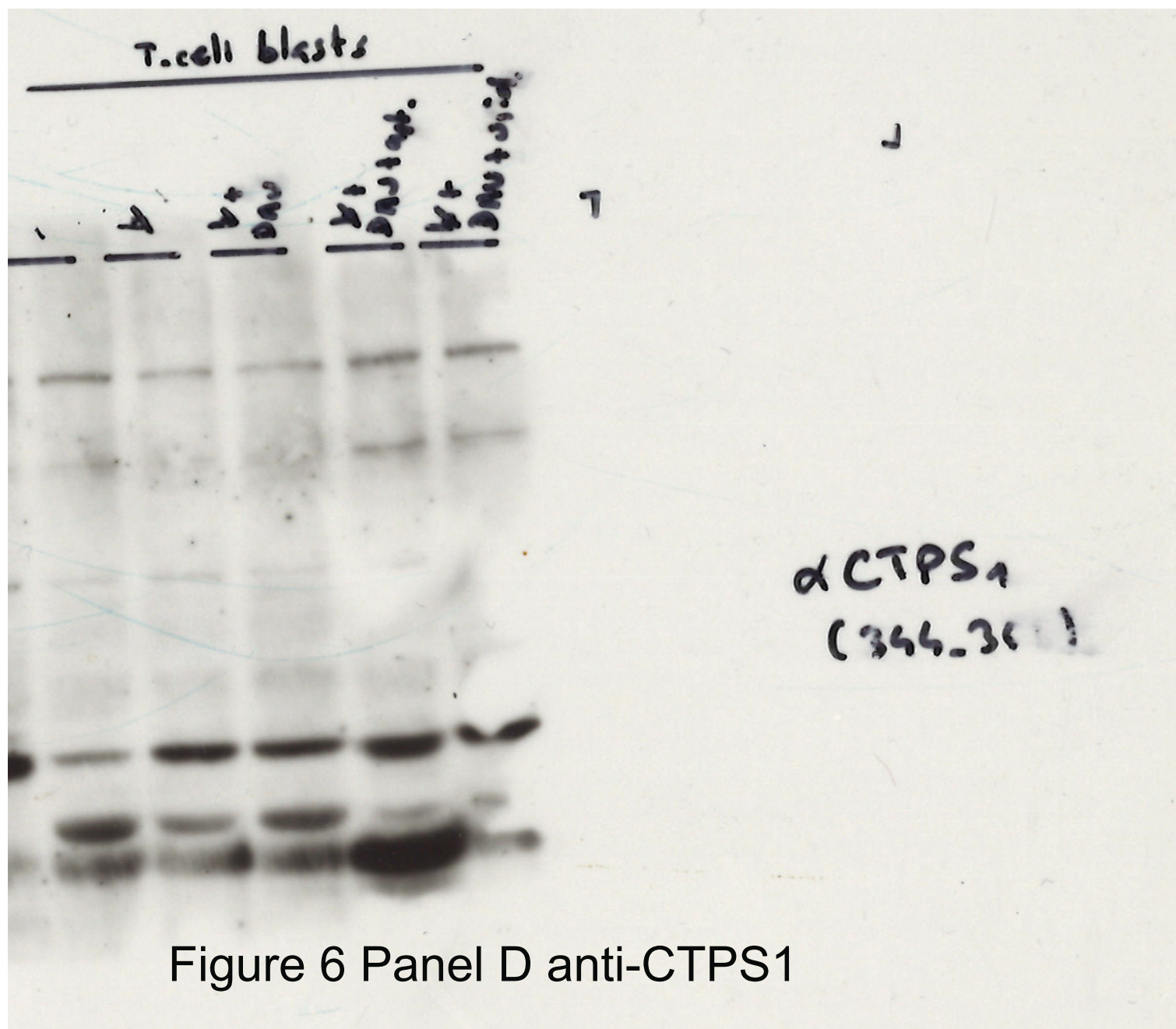

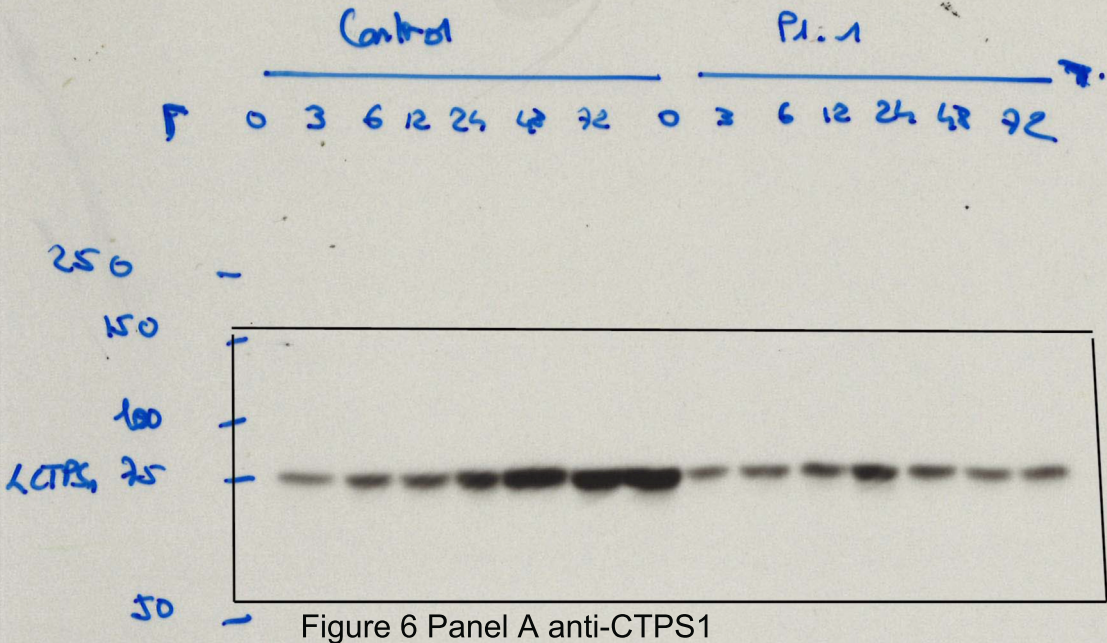

Figure 6 Panel A anti-CTPS1

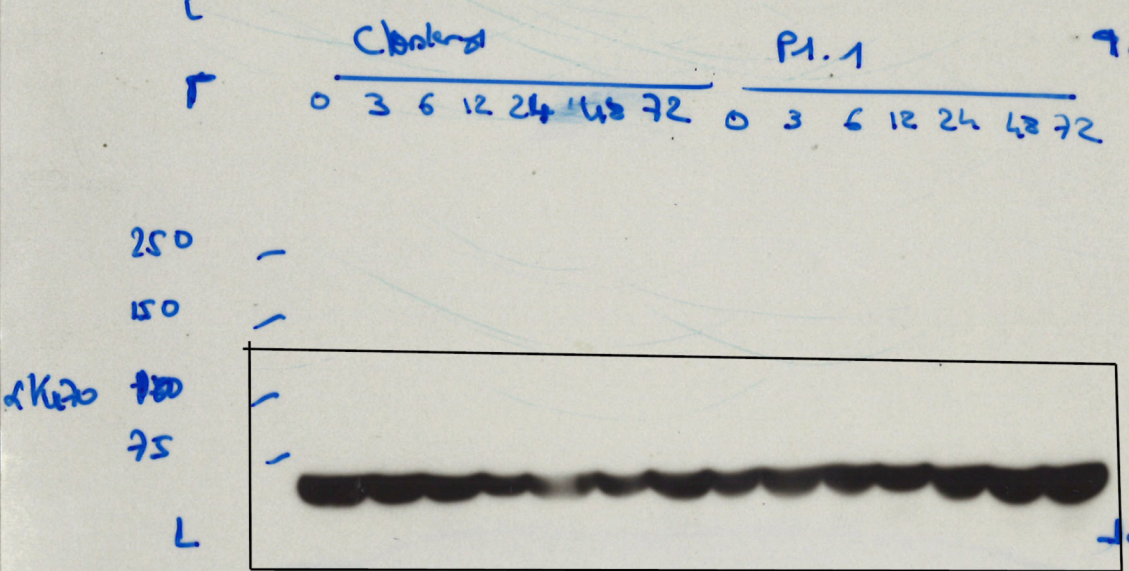

Figure 6 Panel A anti-Ku70

Supplement: Supplementary file 8 — Source Data for Figure 6 [file EMMM-10-188-s006.pdf]
