# Supplementary material for: Loss of RASGRP1 in humans impairs T‐cell expansion leading to Epstein‐Barr virus susceptibility
Source: EMBO Mol Med. 2018 Jan 8;10(2):188–99. doi: 10.15252/emmm.201708292 (PMC5801500; doi:10.15252/emmm.201708292)

Figure 1 PanelF Upper Right anti-RASGRP1

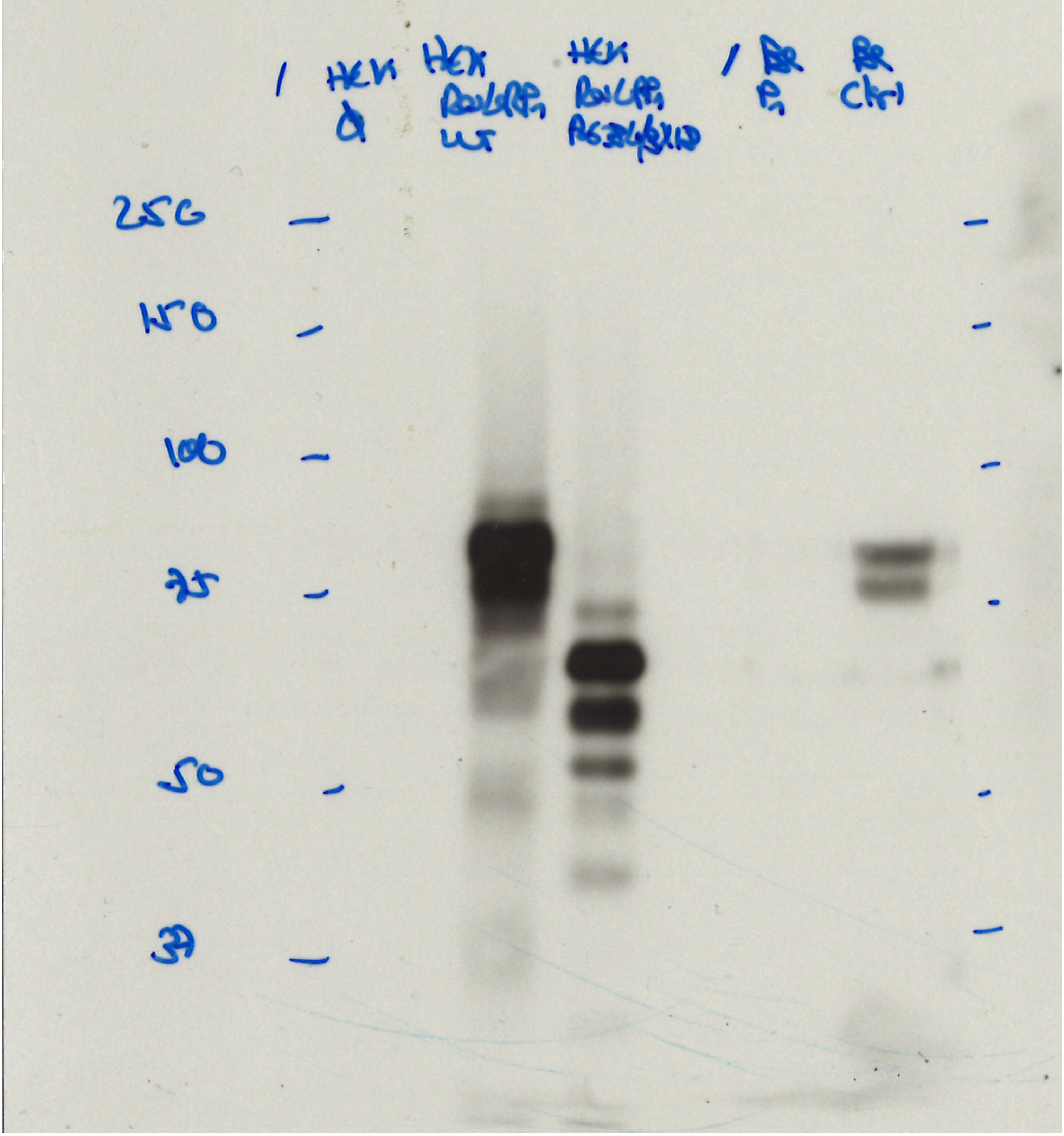

Figure 1 PanelF Lower right anti-ACTIN

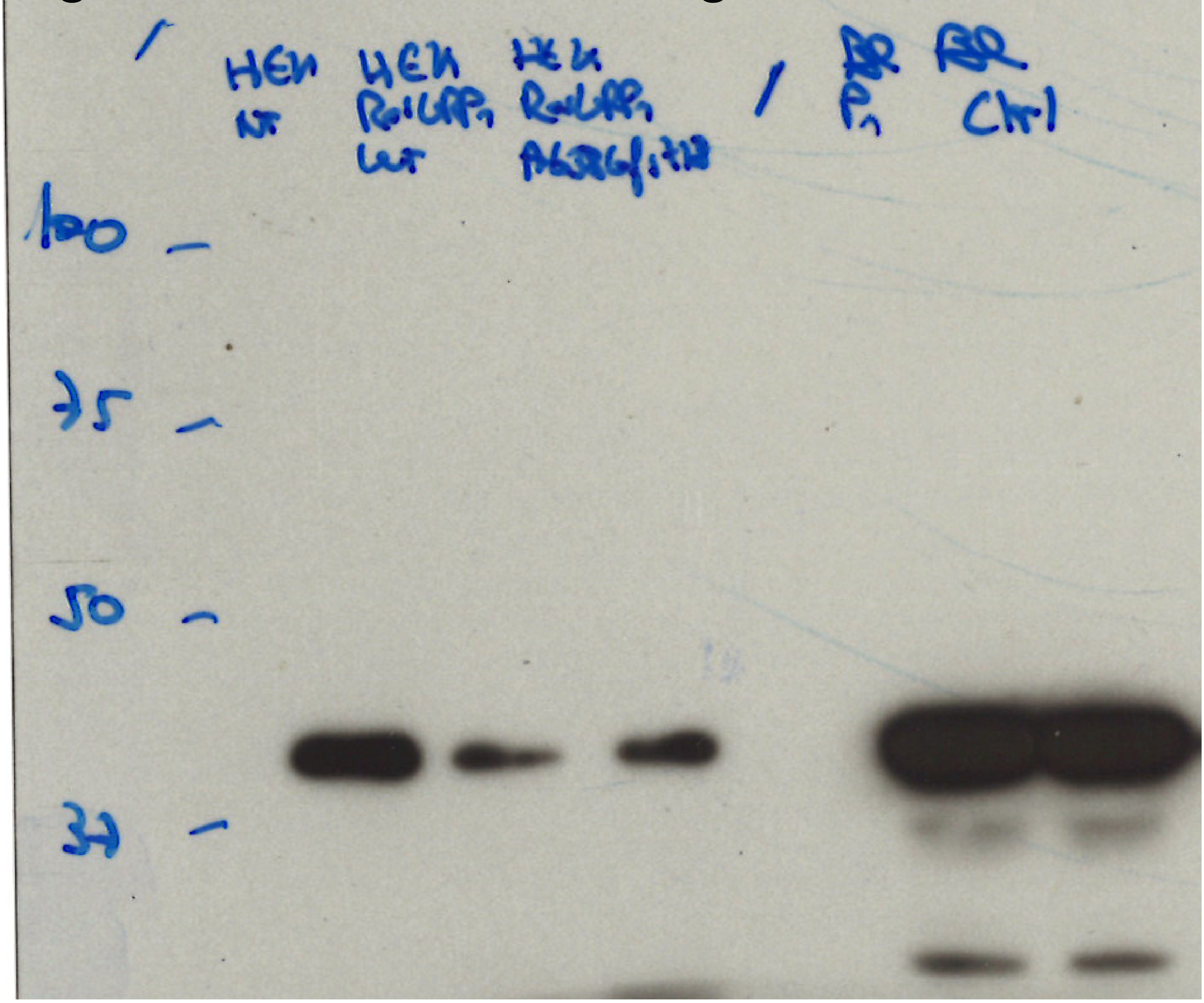

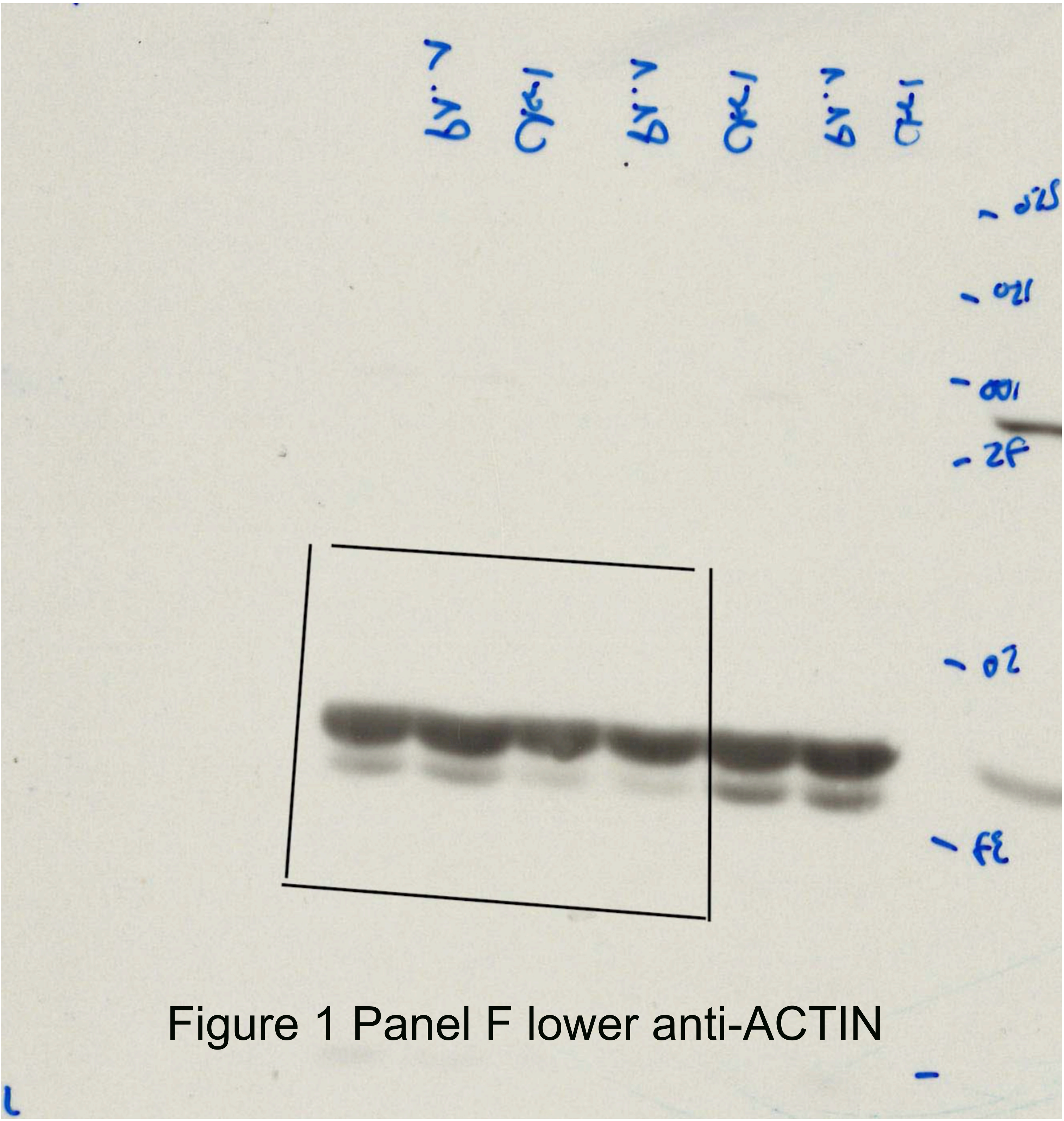

Figure 1 Panel F lower anti-ACTIN

Figure 1 Panel F upper left anti-RASGRP1

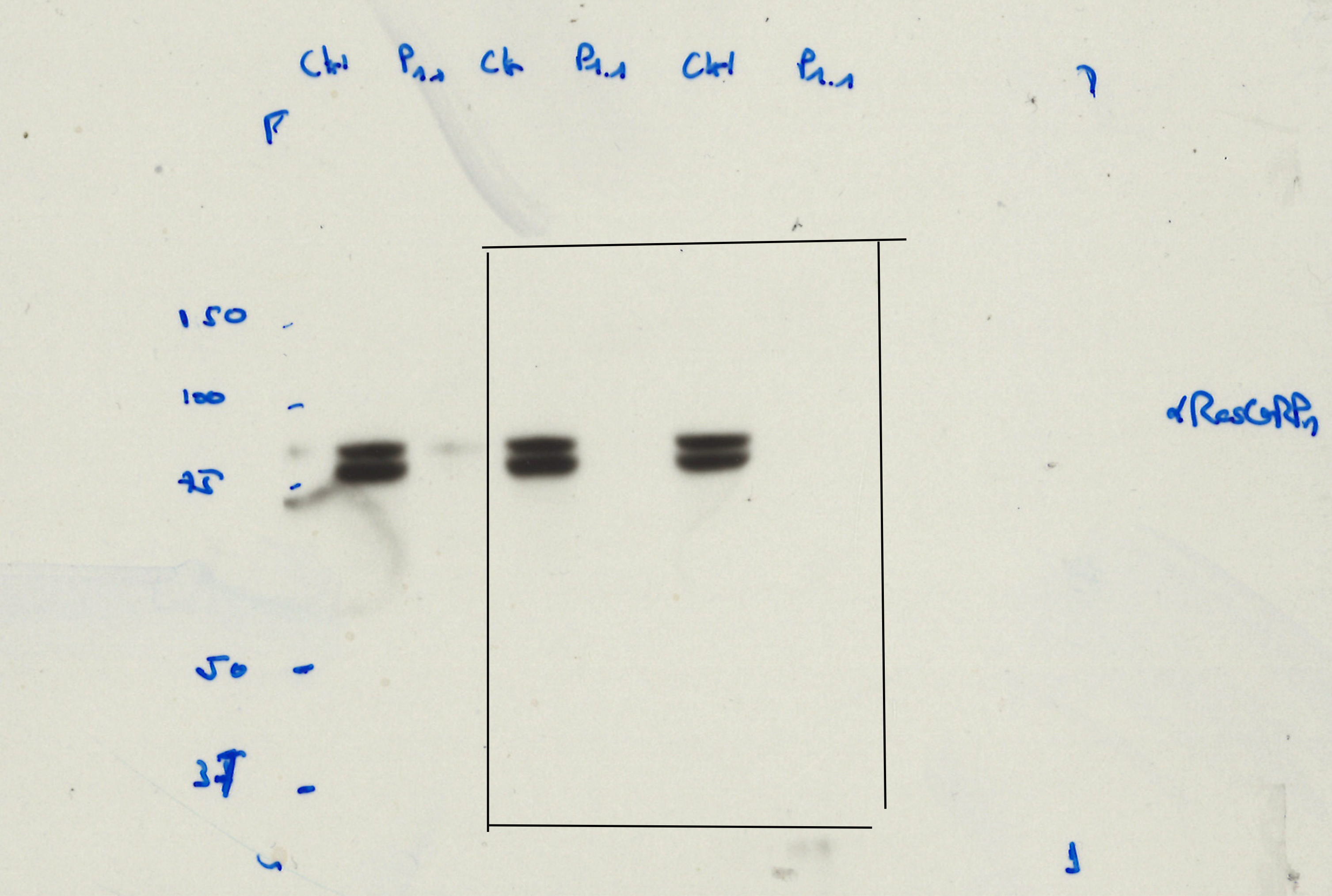

Supplement: Supplementary file 4 — Source Data for Figure 1 [file EMMM-10-188-s002.pdf]
