# Supplementary material for: Loss of RASGRP1 in humans impairs T‐cell expansion leading to Epstein‐Barr virus susceptibility
Source: EMBO Mol Med. 2018 Jan 8;10(2):188–99. doi: 10.15252/emmm.201708292 (PMC5801500; doi:10.15252/emmm.201708292)
Supplement: Supplementary file 5 — Source Data for Figure 2 [file EMMM-10-188-s003.pdf]

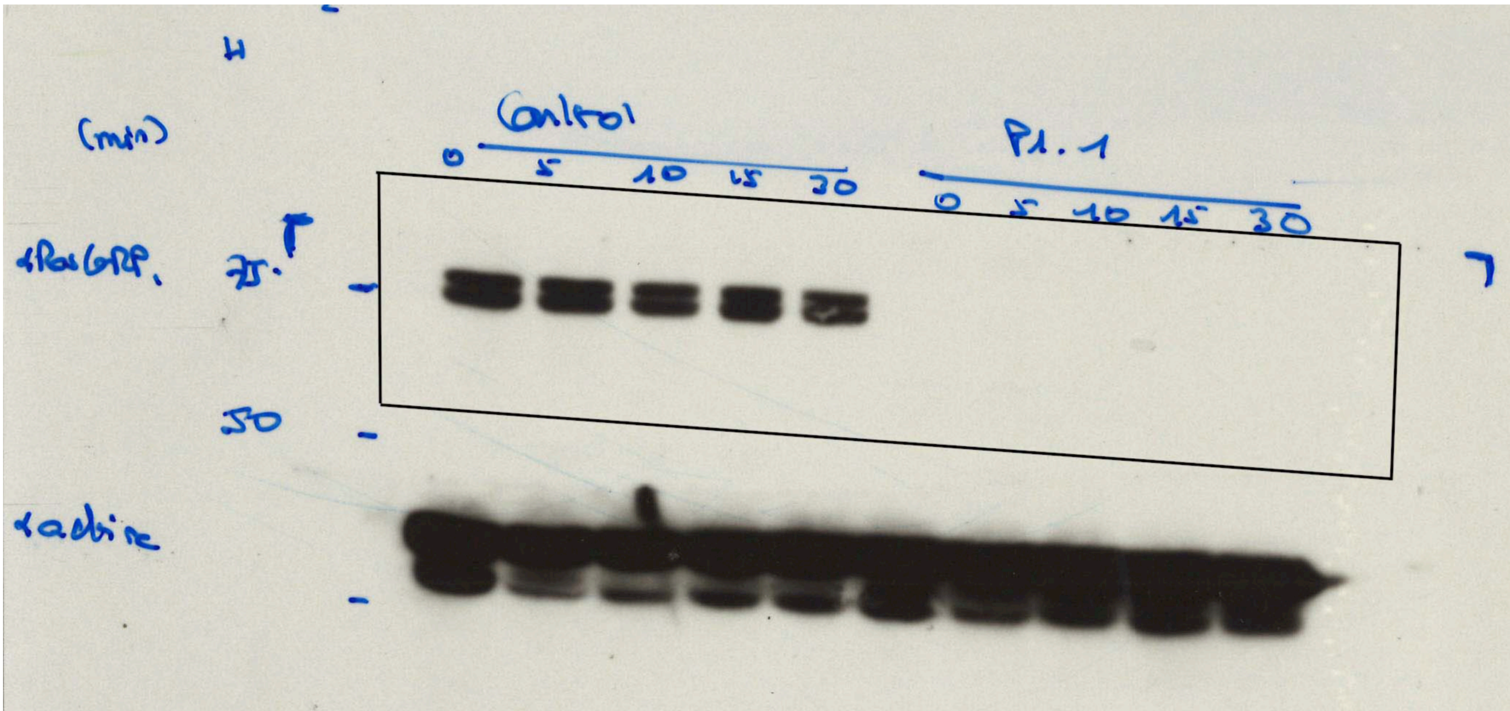


Fig2 Panel C anti-RASGRP1

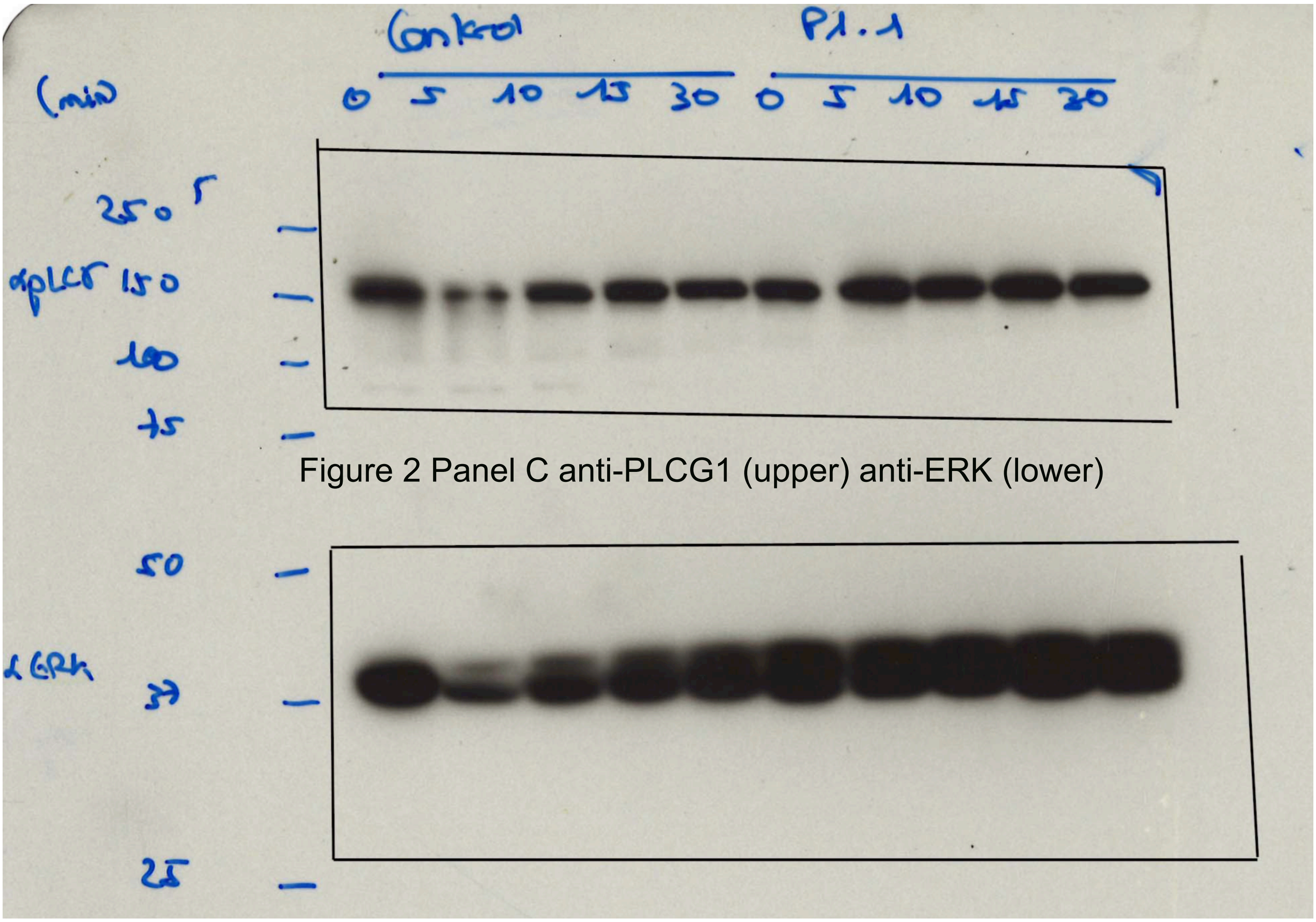


Figure 2 Panel C anti-PLCG1 (upper) anti-ERK (lower)

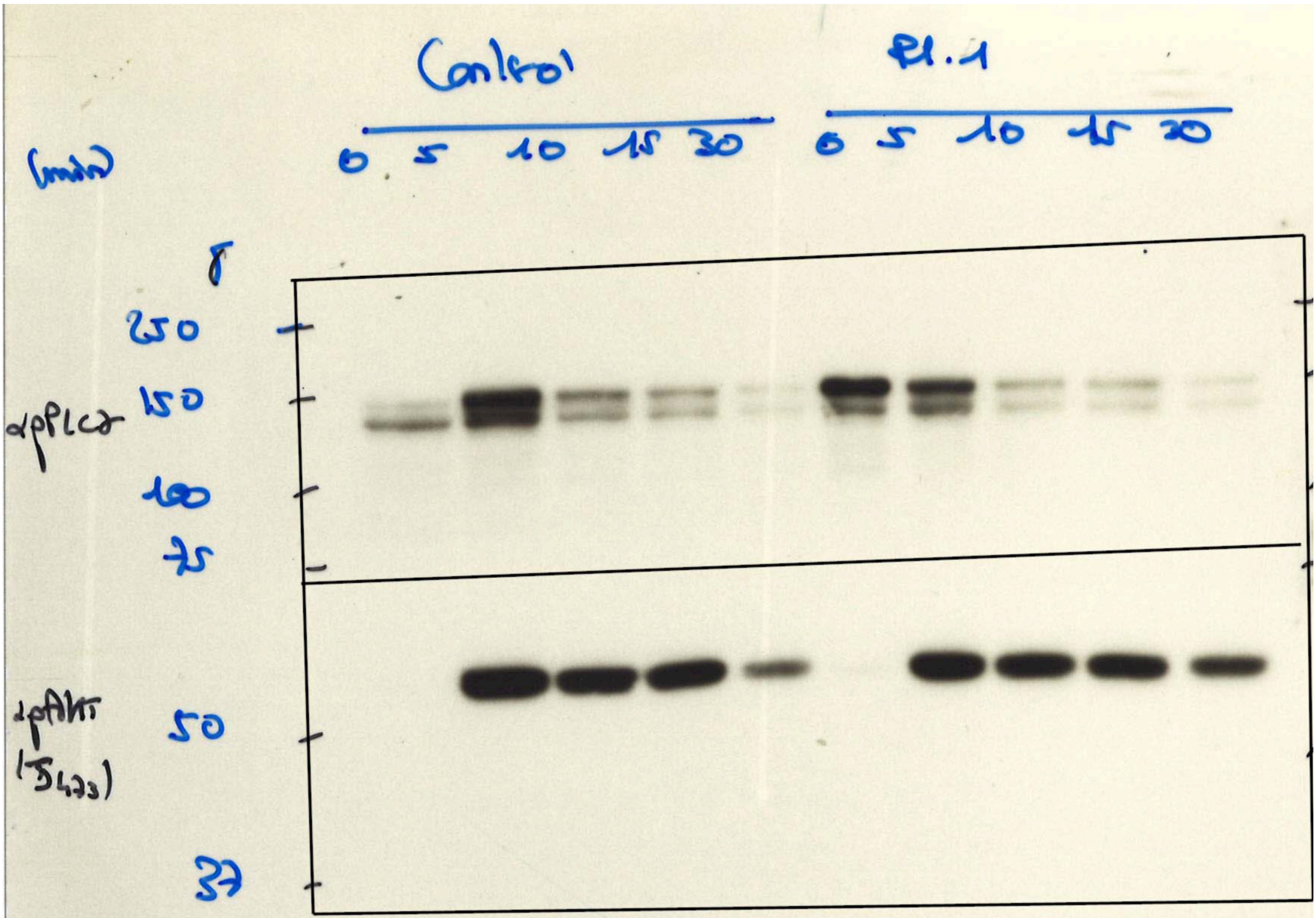


Figure 2 Panel C anti-P-PLCG1 (Upper) anti-P-AKT (Lower)

Figure 2 Panel C anti-P-ERK

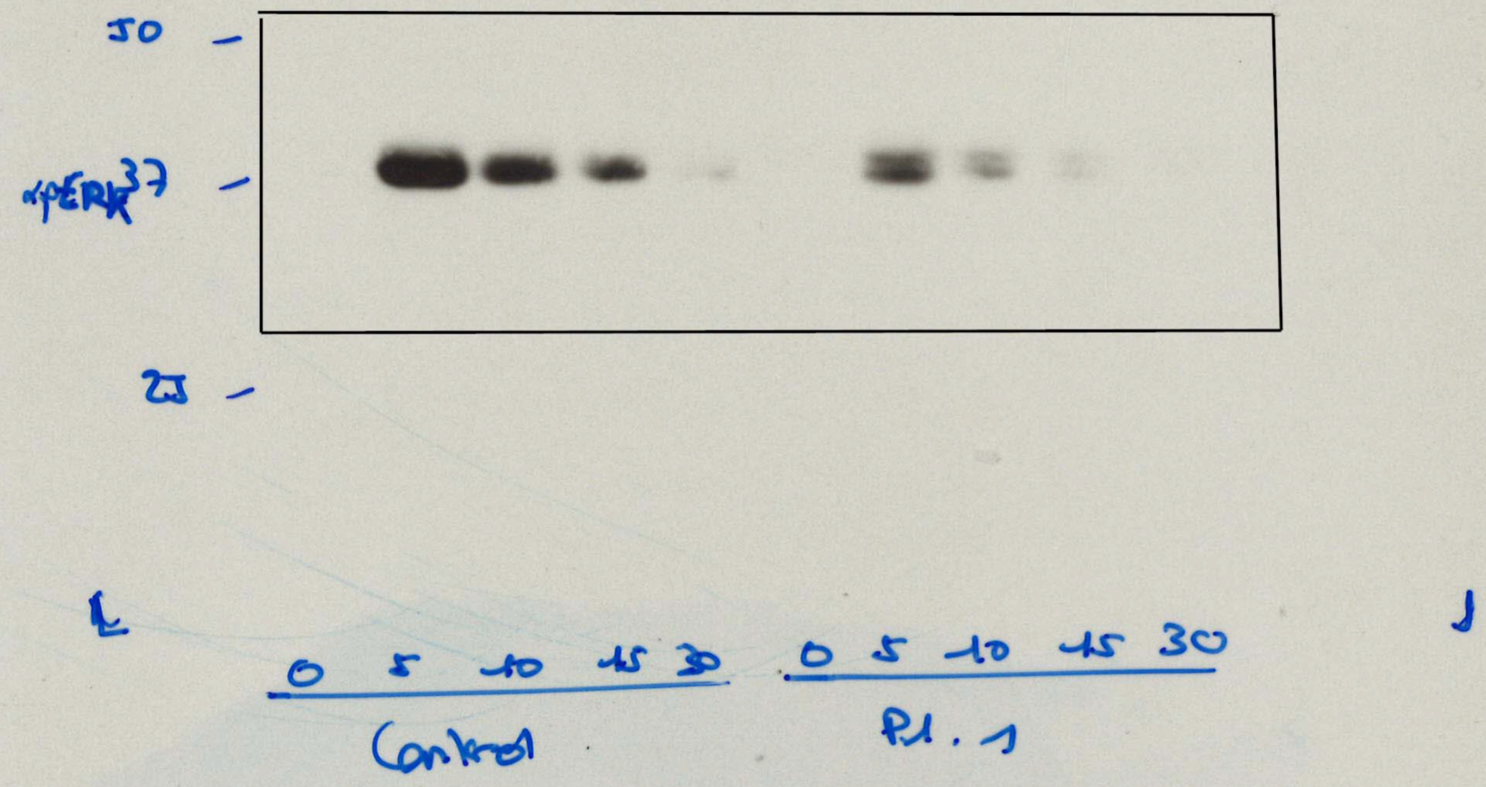

Figure 2 Panel C anti-P-p38

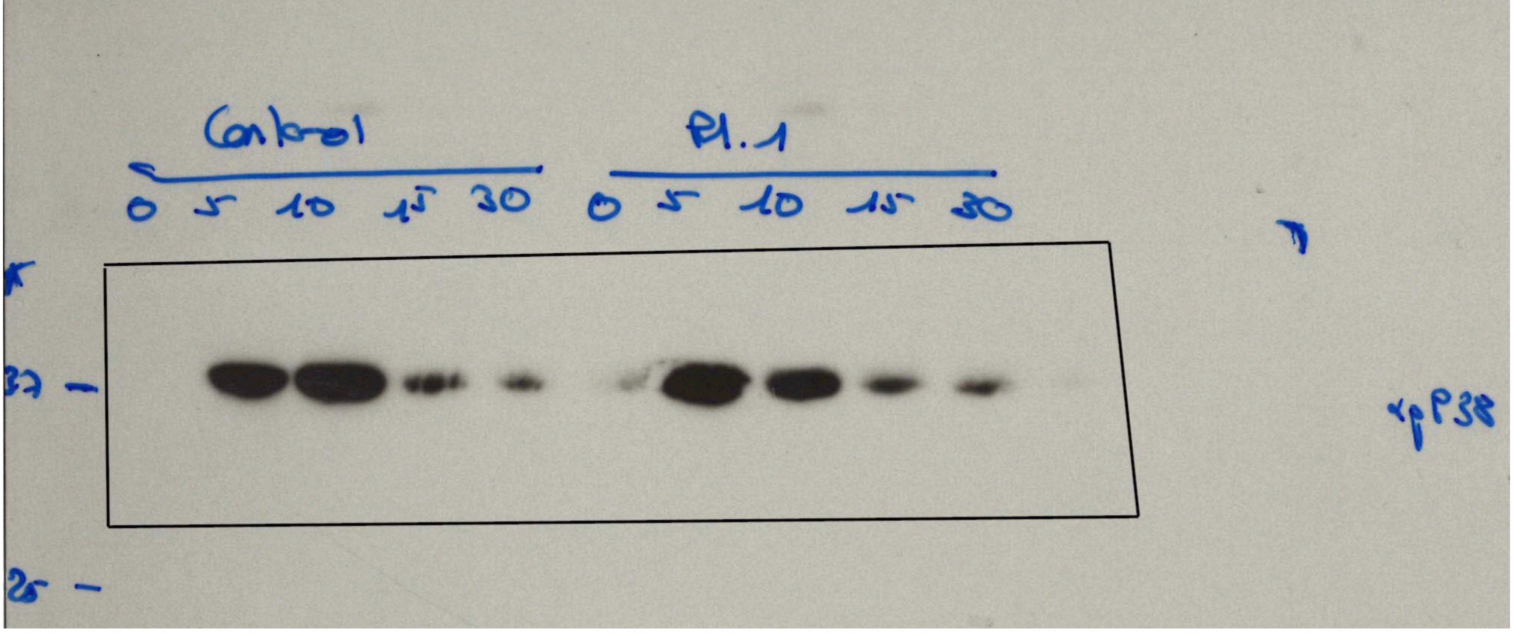

Figure 2 Panel C anti-ACTIN

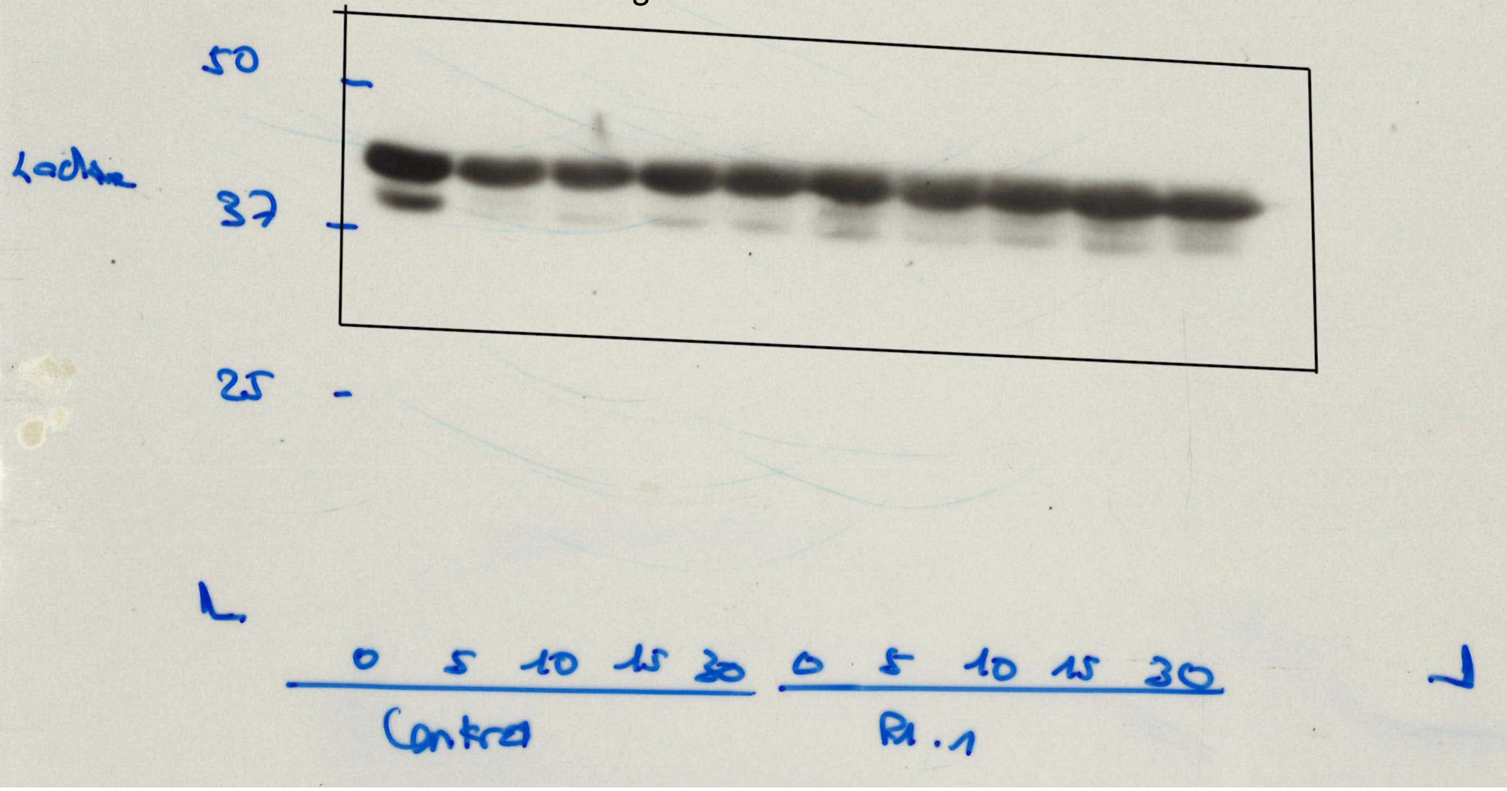

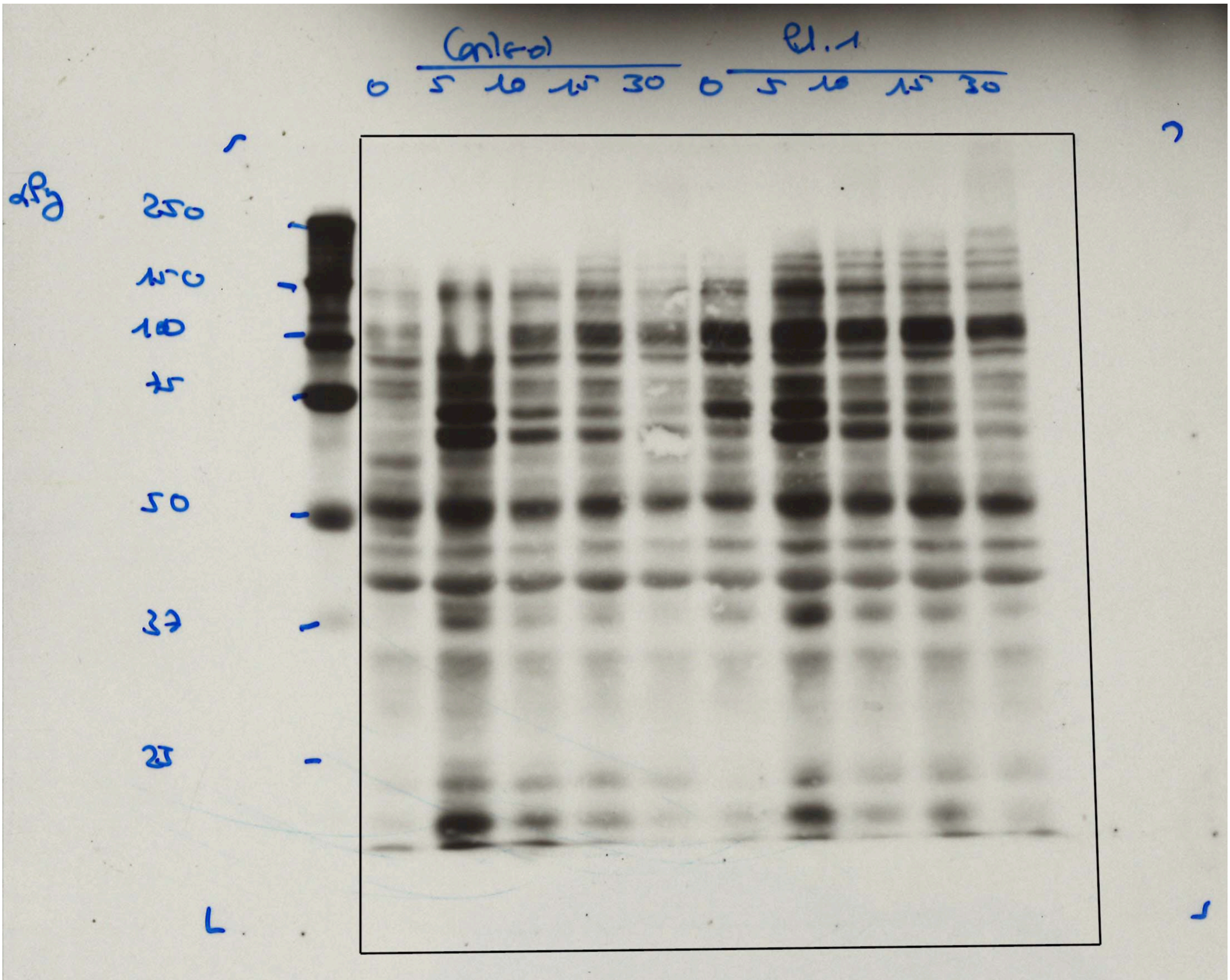


Figure 2 Panel A anti-PY
